# Genetic Geostatistical Framework for Spatial Analysis of Fine-Scale Genetic Heterogeneity in Modern Populations: Results from the KORA Study

**DOI:** 10.1155/2015/693193

**Published:** 2015-07-16

**Authors:** A. N. Diaz-Lacava, M. Walier, D. Holler, M. Steffens, C. Gieger, C. Furlanello, C. Lamina, H. E. Wichmann, T. Becker

**Affiliations:** ^1^Institute for Medical Biometry, Informatics, and Epidemiology, University of Bonn, 53127 Bonn, Germany; ^2^Cologne Center for Genomics, University of Cologne, 50931 Cologne, Germany; ^3^DNA Analysis Unit, Official College of Pharmacists and Biochemists, C1184ABA Buenos Aires, Argentina; ^4^Research Unit of Molecular Epidemiology, Helmholtz Zentrum München, German Research Center for Environmental Health, 85764 Neuherberg, Germany; ^5^Institute of Epidemiology II, Helmholtz Zentrum München, German Research Center for Environmental Health, 85764 Neuherberg, Germany; ^6^FBK, 38122 Trento, Italy; ^7^Division of Genetic Epidemiology, Department of Medical Genetics, Molecular and Clinical Pharmacology, Medical University of Innsbruck, 6020 Innsbruck, Austria; ^8^Institute of Medical Informatics, Biometry and Epidemiology, Chair of Epidemiology, Ludwig-Maximilians-University, 81377 Munich, Germany; ^9^Institute of Epidemiology I, Helmholtz Zentrum München, German Research Center for Environmental Health, 85764 Neuherberg, Germany; ^10^Institute of Medical Statistics and Epidemiology, Technical University Munich, 81675 Munich, Germany; ^11^German Center for Neurodegenerative Diseases (DZNE), 53127 Bonn, Germany

## Abstract

Aiming to investigate fine-scale patterns of genetic heterogeneity in modern humans from a geographic perspective, a genetic geostatistical approach framed within a geographic information system is presented. A sample collected for prospective studies in a small area of southern Germany was analyzed. None indication of genetic heterogeneity was detected in previous analysis. Socio-demographic and genotypic data of German citizens were analyzed (212 SNPs; *n* = 728). Genetic heterogeneity was evaluated with observed heterozygosity (*H*_*O*_). Best-fitting spatial autoregressive models were identified, using socio-demographic variables as covariates. Spatial analysis included surface interpolation and geostatistics of observed and predicted patterns. Prediction accuracy was quantified. Spatial autocorrelation was detected for both socio-demographic and genetic variables. Augsburg City and eastern suburban areas showed higher *H*_*O*_ values. The selected model gave best predictions in suburban areas. Fine-scale patterns of genetic heterogeneity were observed. In accordance to literature, more urbanized areas showed higher levels of admixture. This approach showed efficacy for detecting and analyzing subtle patterns of genetic heterogeneity within small areas. It is scalable in number of loci, even up to whole-genome analysis. It may be suggested that this approach may be applicable to investigate the underlying genetic history that is, at least partially, embedded in geographic data.

## 1. Introduction

Accurate assessment of genetic heterogeneity is relevant to manifold fields, ranging from clinical research, pharmacogenetics, and statistical genetics, over forensic sciences up to evolution (for a review, cf. [1]). In planning genetic epidemiological studies or the collection of control cohorts for prospective studies it is crucial to prevent confounding effects due to undetected or disregarded population structure [2, 3]. Population-based association studies of unrelated individuals, involving case-control and cohort studies, are prone to population structure, which may lead to false positive results or to failure to reveal genuine associations [4, 5]. In family-based linkage analysis unknown population stratification may lower statistical power [6].

Uncovering the genetic basis of complex traits remains an immense and urgent challenge in genetic epidemiological research. Great efforts are set to establish well-designed cohorts and large control samples, intended to serve as basis for genetic epidemiological studies. Besides restricting recruitment to individuals of uniform ancestry, a common strategy applied to efficiently gain a representative sample of the inspected population and to control for potential unknown population substructure is to collect samples in smaller geographical areas, usually in medium to large urban centers (e.g., [7–9]).

Even well-characterized or supposedly homogeneous regions may still account for subtle genetic structure with potential geographical components [3, 10]. Sloan et al. [3] shortly reviewed studies related to geographic genetic structure of human populations and pointed out clear lack of research focusing on genetic heterogeneity of smaller geographic regions or those focused on more urban, highly admixed populations.

Most available well-standardized methods in geographical genetics [11] were developed for other research areas and may not be suited for assessing subtle genetic heterogeneity of modern populations inhabiting geographically restricted areas. Modern humans account per se for the lowest species genetic diversity among primates [12]. A typical western population inhabiting a geographically restricted area sets additional difficulties. Such populations are typically outbred and account for a large degree of admixture, product of older and recent regional, interregional, and even international migration. It is to expect that genetic evolutionary forces, such as selection, mutation, drift, or barriers to gene flow, would play a relatively insignificant role in modeling fine-scale variation of genetic heterogeneity. At this geographical scale, it is more likely that neighborhood preferences and modern mating behavior would have a central role in modeling recent admixture, consequently, having strong influence on the observed pattern of genetic variation of modern small areas (i.e., [13]). In other words, within modern western circumscribed areas, socio-demographic factors would probably explain a large proportion of the observed pattern of genetic heterogeneity.

With the aim of unveiling modest amounts of population substructure in a small, admixed area we (a) searched for subtle patters of genetic heterogeneity and (b) explored potential predictors of the observed patterns. To this end, we combined statistical genetics with spatial statistics (geostatistics) within the framework of a Geographic Information System (GIS). A GIS provides a computational environment designed for spatial analysis of geographic data, therefore the most suitable framework to detect, to model, and to analyze the geographic variation of genetic diversity.

We analyzed a well-characterized cohort collected for prospective studies in a small area of southern Germany. The sampling area included the middle-size city of Augsburg, the surrounding suburban area, and the neighboring countryside. As previously reported by Steffens et al. [14], the KORA S4 sample shows a minimal but measurable increase of the inbreeding factor (8.4*E* − 5% heterozygotes deficit) measured in terms of *F*_IS_ values [15], that is, within-group deviation from expected heterozygosity but no indication of population substructure. Despite extensive search of potential population stratification with the software package STRUCTURE [16], in this cohort no signals could be detected [14]. The STRUCTURE program implements a model-based clustering method. It estimates the proportion of individuals' genome that may originate from differential populations, the probability that an individual belongs to a certain population as well as allele frequency differences in terms of Wright's *F*_ST_ statistics [17]. Regardless of intensive computations under several models, STRUCTURE results did not provide any indication of a potential pattern of genetic heterogeneity in the KORA S4 survey [14].

## 2. Material and Methods

### 2.1. Subjects and Genotypes

Our analysis is based on a subset of the KORA cohort (Kooperative Gesundheitsforschung in der Region Augsburg; in English: Cooperative Health Research in the Region of Augsburg; [7, 9]). The KORA survey is an ongoing study, which takes place in a circumscribed region of southern Germany: Augsburg City and the two neighboring districts. The KORA cohort was recruited for prospective studies. In 4 surveys (S1–S4), a total of 18,000 participants were randomly selected from the adult population of resident German citizens (25–74 years) [7, 9]. Phenotypic, socio-economic information, and residence locality were gathered. The KORA cohort is a sample of the extant German population in the region.

The analysis was conducted on a random set of the KORA S4 survey (*n* = 4261), recruited in the period between the years 1999 and 2001 [7, 9]. The data set consisted of 728 unrelated healthy German citizens, which included subjects born within and outside of Germany as well. The graphical method GRR (Graphical Representation of Relationships; [18]) was used to exclude the presence of biological relationship of individuals based on genetic data (see Supplement 7). In this paper we distinguished the portion of Germans citizens born outside of Germany as “immigrants” and those born in Germany as “natives.” The immigrant group (*n* = 179) included subjects born in twenty worldwide distributed countries, half of these countries represented only once. Four countries, Czech Republic, Romania, Poland, and Ukraine, corresponded to the land of birth of 82 percent of all immigrants. The group of subjects born in one of these four countries was classified as “major immigrant group.” Based on the information “land of birth” we differentiated between data sets: (a) ALL: the complete data set of 728 subjects (resident German citizens), (b) GER: the total set of 549 natives, and (c) MAIN_IMG: the subset of 146 immigrants, born in either Czech Republic, Romania, Poland, or Ukraine.

The KORA S4 sample was genotyped for 212 single nucleotide polymorphisms (SNPs) (Supplementary Table  S6 in supplementary material available online at http://dx.doi.org/10.1155/2015/693193) [14]. These SNPs can be differentiated in two sets. The first set includes 68 coding SNPs located in exons of functional genes. These SNPs either cause an amino acid exchange or an effective promotor alteration in respect to the resulting protein. Assuming evolutionary times these SNPs may be subject to selective forces. The second set comprises 144 neutral SNPs. These loci were chosen at random throughout the genome in putative “genomic deserts,” pursuing to achieve uniform distribution across the genome (setting a minimum of 500 Kbp intermarker distance). For this selection only SNPs presenting a minor allele frequency between 10 and 50% in Caucasians were considered. The markers included in the final intergenic set were uniformly spaced and located >100 Kbp apart from any known genes and >1 Mbp apart from centromeres and telomeres. This procedure followed the set of rules proposed by Devlin and Roeder for genomic control markers [19]. Accordingly, these intergenic SNPs are assumed to be neutral to selection forces in the absence of any specific information. In this sense, these loci are expected to reflect the effects of demographic processes involving migration (gene flow) and even drift, if evolutionary times are considered. Steffens et al. [14] undertook an extensive quality assessment to this data set. The averaged call rate over all samples was 97.3%; intragenic SNPs achieved an average call rate of 96.2% and intergenic SNPs, an average call rate of 97.9% [14]. Details of the genetic properties of the full set of 212 SNPs are listed in Supplementary Table  S6.

### 2.2. Study Area

**Figure 1:**
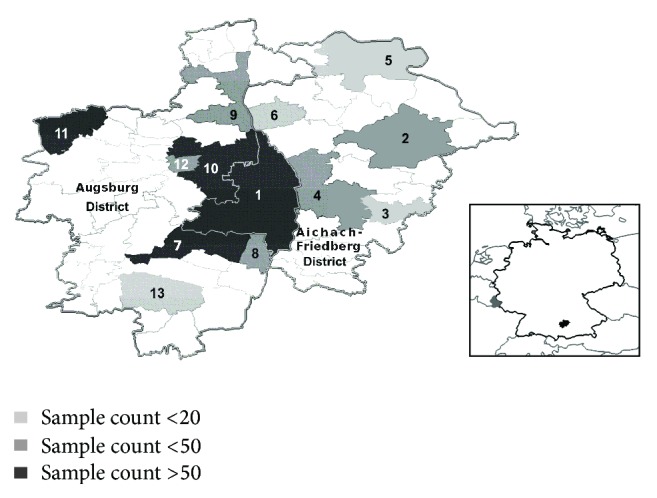
Study area. Land units: (1) Augsburg; (2) Aichach; (3) Eurasburg; (4) Friedberg; (5) Pöttmes; (6) Rehling; (7) Bobingen; (8) Königsbrunn; (9) Langweid (Meitingen); (10) Neusäß (Gersthofen; Stadtbergen); (11) Altenmünster; (12) Aystetten; (13) Schwabmünchen. Location of the study area within Germany is indicated in the inset in black; German boundaries are displayed with a black line, neighboring-country boundaries are displayed with a gray line; Luxembourg, a country covering an extension similar to the study area, located on the western boundary of Germany, is displayed in gray.

The study area comprised three administrative regions: the municipality of Augsburg City and its two neighboring districts, Aichach-Friedberg District and Augsburg District (Figure 1). It covered an area of approximately 2,970 km^2^. This is a surface comparable with the Grand Duchy of Luxembourg (Figure 1). The area is located in the Swabia administrative region of Bavaria, southern Germany, between the coordinates 10.491°E/48.091°N and 11.310°E/48.642°N. The population had approximately 630,000 members in 2004. The mean population density is 212 inhabitants/km^2^, a figure that is comparable with the German average.

Augsburg City is a typical middle-size German urban area. The Aichach-Friedberg and Augsburg Districts include a suburban area neighboring Augsburg City and a periurban area, a patchy pattern of smaller cities and villages widespread across a rural landscape.

### 2.3. Regionalization Methods

The spatial analysis required diverse types of regionalization of the study area. Three regionalization methods were applied: (a) a subdivision of the total study area into minimal representative spatial units of analysis; (b) a subdivision of the total study area into contiguous sampled units using a polygon-based method; and (c) modification of the first regionalization in order to achieve a set of contiguous spatial analytical units while retaining the original geometry defined in (a).

#### 2.3.1. Land Units

Genetic landscapes, in this work referred to matricial representations of genetic variation in the geographic space, were created with geostatistic methods of surface interpolation (see Section 2.7.1). For this objective, it is convenient to define a minimal spatial unit of analysis which is representative of data coverage and it covers a spatial surface much smaller that the phenomenon of interest.

The basic spatial unit of analysis of the genetic landscapes was the postal area. The postal area corresponds to the smallest district or region defined by the German postal system (the German postal system divides Germany in ca 28.700 postal areas). We considered this an appropriate analytical area because the German postal system divides the territory into spatial units with a similar number of inhabitants, independent of the extension of the spatial unit. Similar population size among land units allows adequate comparisons from socio-demographic perspective. Postal areas include as well a population size large enough in order to guarantee subjects' anonymity. A finer geographical reference of subjects, that is, postal address, was not available and it would not be in agreement with local official restrictions in respect to personal anonymity. We considered the subdivision of the study area into postal areas adequate to identify and to analyze fine-scale patterns of genetic variation. The study area included a total of 64 postal areas. The spatial extension of the postal areas ranged from 1.8 km^2^ to 93 km^2^, with an average of 26 km^2^.

The sampled area covered about 20% of the total study area, that is, approximately 600 km^2^ (Figure 1). It included Augsburg City and 15 settlements located in Aichach-Friedberg District and Augsburg District. Each sampled settlement corresponded to one postal area, except for Augsburg City. Augsburg City itself contains 14 postal areas. In summary, out of a total of 64 postal areas, data was available in Augsburg City (including 14 postal areas) and in another 15 postal areas. Augsburg City samples were pooled together for frequency computations, since no information about postal area of residence was available for residents in this city. A subdivision of Augsburg City into postal areas was only considered in the step of spatial interpolation to improve interpolation results (see Section 2.7.1). Postal areas with a very low number of samples were aggregated to neighboring sampled areas in order to exclude bias due to low number of samples per land unit. Explicitly, the quarters Stadtbergen (*n* = 7) and Gersthofen (*n* = 6) were aggregated to Neusäß (*n* = 47); Meitingen (*n* = 12) was aggregated to Langweid (*n* = 22) (Figure 1). In the final geostatistical analysis the sampled area included 13 analytical land units. In this work analytical land units (areal representing sampled data) defined on the basis of the geographical coverage of postal areas are further referred to as* land unit* (LU). LUs were labeled with the sampling-location name; aggregated LUs were labeled with the name of the location accounting for the largest number of samples. Augsburg City included the maximum number of samples (*n* = 359). The remaining 15 sampled postal areas (aggregated into 12 LUs) included a total of 369 samples. Letting aside Augsburg City, sample size per LU ranged between *n* = 9 samples (Eurasburg) and *n* = 60 samples (Neusäß). The mean sample size per LU was 30.8 and the standard deviation was 17.4 (Table 1).

#### 2.3.2. Polygon-Based Regionalization

The implementation of the spatial autocorrelation tests performed in this study (see Section 2.7.2) required to count with a set of adjacent spatial analytical units. This means that only spatial units with at least one contiguous neighbor could be included in the analysis.

**Figure 2:**
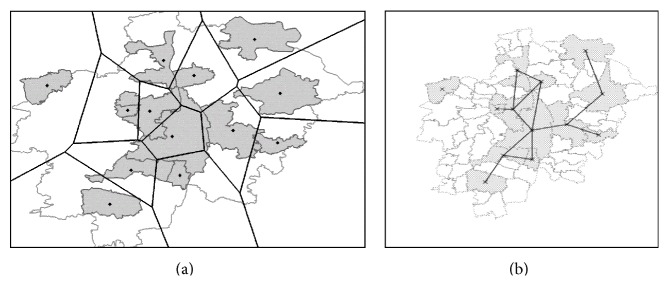
(a) Regionalization of the study area in 13 Thiessen polygons; each polygon represents a LU. LU centroids were used to delimit the Thiessen polygons; (b) net of contiguous LUs; three LUs were spatially connected to the next sampled LU performing a geometrical correction of LUs' boundaries: Schwabmünchen was connected to Bobingen, Aichach to Friedberg, and Pöttmes to Aichach; vectors show pairs of LU assigned a spatial weight equal to unity in the matrix of spatial weights; pairs of LUs not connected with vectors received a value equal to zero in this matrix.

The total study area was divided into 13 Thiessen polygons (designation given to Voronoi diagrams used to analyze spatially distributed data) [20]. Each polygon corresponded to one LU defined in the first regionalization (see Section 2.3.1). We chose this simple type of regionalization since many natural patterns may be closely approximated to this type of areal structure. Thiessen polygons were delimited based on the centroids (polygon geometrical center) of the 13 sampled land units (Figure 2(a)). The Voronoi tessellation was created with the method  v.voronoi  of the open-source software package GRASS 6.4 (Geographic Resources Analysis Support System, http://grass.osgeo.org/).

#### 2.3.3. Net of Contiguous Sampled Units

The implementation of the algorithms used in this study to search for best predictors of spatial variation fitting the data (see Section 2.7.3) required as well contiguous analytical units. For such more complex analysis, the coverage of each LU was retained. In this case the first step was to verify the presence of direct neighbors for all LUs. Four LUs did not account for contiguous neighbors: Aichach, Pöttmes, Schwabmünchen, and Altenmünster (Figure 1). Of these, the first four LUs were not further than 5 km away from the next closest LU border. This geographical distance was considered negligible in the context of connectivity and human interaction between modern settlements. In order to get maximal information of the available data we modified slightly the geometry of these four LUs and of their closest neighbors in order to meet the contiguity condition for at least 12 LUs. Schwabmünchen was connected to Bobingen, Aichach to Friedberg, and Pöttmes to Aichach (Figure 2(b)). With this step, 12 LUs conformed a continuous geographical space. The most peripheral sampled land unit, Altenmünster, without a close sampled contiguous neighbors (distance to the closest LU > 10 km), was not included in the computations (Figure 2(b)).

#### 2.3.4. Matrix of Spatial Weights

Both implementations of spatial dependence analysis performed in this study required the definition of a matrix of spatial weights representing the interaction between LUs (see Sections 2.7.2 and 2.7.3).

On the basis of the previously defined regionalization, either the Thiessen polygons (Figure 2(a)) or the geometrically modified LUs, for each analytical unit the geographical central point (the* centroid*), were specified. These two tests were performed in this study based on a binary representation of the spatial weight matrix, which assigned a weight of unity for neighbors, and zero otherwise. A binary encoding was chosen since not enough information was available to set assumptions about the assumed spatial process. The function  poly2nb  was used to construct the neighbor list with default parameters and the function  nb2listw to construct the weight matrix, setting the function parameter  style  = **B**  for a binary system. Pairs of LUs assigned a spatial weight equal to unity are indicated in Figure 2 with a vector net. All other pairwise combinations of LUs received a spatial weight equal to zero.

### 2.4. Socio-Demographic Parameters

Socio-demographic information collected during recruitment included* age*,* education years*,* degree of professional training and education*, and* place of birth*. Age ranged between 25 and 74 years. Education years ranged between 8 and 17 years old. Detailed descriptions of demographic features are provided in Supplementary Table  S1.

As described in the Introduction the KORA S4 survey mirrors the case of plenty of study designs in human genetic research, in which control cohorts are used for population studies. In the context of these studies it could be crucial to assume genetic homogeneity of controls. One strategy is to collect samples in small areas and to restrict recruitment to individuals of same ancestry (see Section 1). In the concrete case of the KORA S4 it could be verified that the presence of immigrants introduced a small but significant effect on the total amount of genetic variation (see Supplement 4). In this paper we focus on the case of a population that may be considered* a priori* to be genetically homogeneous and may account for subtle genetic substructure and if this is the case, which factors may be regarded as best predictors. To perform our study in accordance with these objectives and assumptions we worked with the two data sets. We first considered the total sample (ALL). We used this set with the purpose of inspecting the effect of immigrants on the total genetic variation among other factors. On the other side we excluded immigrants and analyzed the subset of natives (GER). This analysis is intended to specify best predictors of sublet genetic substructure and to estimate their effect in an admixed modern small population. The total set of immigrants did not include enough individuals of similar ancestry to perform further separated geostatistical analysis. Therefore no further group with homogeneous ancestry could be identified.

Measures related to age, education years, and education level were computed only on the native GER data set. Variables related to immigrant representation in the total sample were computed for the total data set (ALL). All measures were computed per land unit.

(I) Variables related to age, education years, and education level, computed only for the GER data set:

 AGE25_39: percentage of subjects in the age category of 25 to 39 years;
 AGE40_54: percentage of subjects in the age category of 40 to 54 years;
 AGE55_74: percentage of subjects in the age category of 55 to 74 years;
 AGE_MEAN: mean age;
 EY8_11: percentage of subjects achieving a maximum of 11 school years; this variable indicates the fraction of the sample which did not achieve the education level required to access to academic studies;
 EY_MEAN: mean years of school attendance;
 EDU_MEAN: mean education level, scored according to the degree of professional training and education, ranging from 0 = no school degree up to 9 = graduate degree (M.S. equivalent or higher).

(II) In order to inspect the effect of immigrant representation on total population from a model building perspective, variables related to birth land were included; these variables were computed for the ALL data set.

The representation of the total immigrant fraction in relation to the total sample was modeled with the variable:

 GER_P: percentage of natives over all subjects.

(III) on the same line and for purpose of ascertaining a potential effect related to the presence of major fraction of immigrants incoming from a reduced number of countries, which could be acting as a differentiated population within the migrant group, a further variable was included:

 MAIN_IMP: percentage of the major group of immigrants (subjects born in Czech Republic, Romania, Poland, or Ukraine) over all immigrants (German citizens born outside of Germany).

### 2.5. Measure of Genetic Diversity per Land Unit

We attempted to achieve a reduction in form of genetic landscapes of the complex georeferenced data available for this cohort (genotype per SNP and sample and geocoordinates of LUs). These genetic landscapes should allow visualization of the estimated distribution of genetic diversity across geographic space and further assessment of associations between spatial patterns of genetic diversity and other georeferenced data, for example, average socio-demographic characteristics per LU.

We chose to create maps based on indices calculated per LU. The reason for this is that we considered these types of genetic landscapes easier to interpret than those based on relative measures, for instance, the genetic differences between LUs. For this step it was necessary to select a genetic measure of diversity referred to each single LU, in opposition to relative measures such as Wright's *F*_ST_ [17] or alike, which would characterize variation of genetic diversity in terms of pair of LUs.

The average heterozygosity is a usual measure of the genetic variability of a group [21]. We chose this simple measure of genetic diversity, the observed heterozygosity (*H*_*O*_) [22], to summarize sample genetic attributes of each LU. *H*_*O*_, the observed frequency of heterozygotes averaged over loci, was estimated using

(1) $$\begin{array}{c} H_{O}=\frac{\sum_{j}^{l}h_{j}}{l}, \end{array}$$

where *l* is the number of loci and *h*_*j*_ indicates the proportion of heterozygote individuals per locus *j*_th_ [22]. Observed heterozygosity was computed separately for each land unit with the total data set (*H*_*O*_(ALL)) and with the subset of natives (*H*_*O*_(GER)).

### 2.6. Multivariate Analysis of Spatial Population Structure

In an attempt to frame the challenge embedded in this sample, further genetic measures were computed with well-standardized tools for detecting population structure. First exploratory analysis with geostatistical methods indicated a potential differentiation of the periurban areas from Augsburg city and its periphery (see Supplement 2). Potentially, the fine-scale patterns of genetic diversity observed in these first exploratory evaluations could be explained by various simple models of spatial variation. For instance, the observed pattern (Supplementary Figure  S2a-b) may be the result of a simple process of isolation by distance. As a result, genetic diversity would follow a pattern of gradual variation (e.g., gradients of allele or genotype frequencies). It must be noted that in such case, the observed pattern would correspond to a small fraction of the geographical landscape where the process occurs. This is so because both geographic extension of the study area and evolutionary times of the study population (here it refers to the number of generations necessary to fix the effects of any gene-flow process [23, 24]) are jointly, most probably, not large enough to have generated a local process of fixation of gradual variation of genetic features. Spatial correlation methods (e.g., spatial autocorrelation) and Mantel tests would be the first methods of choice to detect spatial correlations of genetic distance with geographic distance.

The observed pattern (Supplementary Figure  S2a-b) could as well be the product of undetected population clustering. In this case, individuals of similar genetic features tend to reside in distinct areas than individuals less similar. Clustering would also require that individuals of distinct groups present reduced interaction with individuals of other groups. At larger geographical scales this could be observed when cultural, linguistic, or political limits set a barrier to gene flow. It is important to note that this scenario is less probable. For instance, it is improbable that a modern western population inhabiting such a small area would be composed of several groups with reduced exchange (low migration rates among the subareas and low predisposition to mate with individuals of other groups). This situation is even less probable if considered that spatial patterns of genetic variation were even detected within the group of natives. The result is supported by previous analysis undertaken with this sample: an exhaustive evaluation of population clustering was conducted by Steffens et al. [14] with the well-known software STRUCTURE [16]. Despite the large number of runs with varying models and parameters there was no indication of any population substructure. Results indicated that the model assuming a number of populations equal to unity (*K* = 1) showed the highest posterior probability for the KORA S4 data.

The following software packages were used in this step: (a) GENELAND [20, 25]; (b) EIGENSTRAT | EIGENSOFT [26, 27]; (c) PLINK, version v0.99s (http://pngu.mgh.harvard.edu/purcell/plink; [28]), with additional multidimensional scaling using the R software package, version 2.12.1 (R Foundation for Statistical Computing, 2010); (d) SPAGeDI [29]. The wide-spread used software STRUCTURE [16] was not considered, since in a previous study [14] no evidence for genetic substructure was found with this tool. Methods (a) to (c), as well as STRUCTURE, share the possibility to search for groups of genetically similar individuals. SPAGeDI (d) is a tool for detecting dependency between genetic and geographic distances among individuals or populations. GENELAND (b) and SPAGeDI (d) are individual-based methods and require including in the computation the geographic reference of each individual. As mentioned above (see Section 2.3.1), available data and anonymity restrictions did not allow a more precise georeference of subjects than sampling location. For these reasons, all individuals sampled in one location were georeferenced to the same geographical coordinates. This data aggregation consequently involves loss of power when applying these methods. Therefore, in our case and as it most probably would occur in this type of human genetic studies, the full capabilities of software making use of individual geographical coordinates could not be exploited.

With each tool (a–d), several exploratory runs were performed. In each case runs were started with default parameters and recommended model assumptions. Following, multiple runs with varied parameter values and model assumptions were conducted. For computations demanding* a priori* definition of an assumed number of subpopulations, runs were repeated for incremental number of subpopulations not larger than ten.

### 2.7. Geostatistical Analysis

Geostatistical analysis was conducted using the open-source software package GRASS 6.4 and spatial packages contributed to R software package, version. 2.12.1 (R Foundation for Statistical Computing, 2010) within the GRASS environment.

#### 2.7.1. Generation of Genetic Landscapes

In this framework, genetic landscapes were defined as matrix representations of genetic variation in the geographic space. Spatial matrices were created by the transformation of sampling-point data to an elevation surface by spatial interpolation. An elevation surface is a 3D layer of continuous data (grid or raster layer) with elevation information at each point of the area. GRASS defines this type of spatial object as 2.5 dimensions (2.5D). As a simplification, the usual denomination for this type of spatial object: “3 dimensions (3D)” is adopted. The elevation parameter characterizes the estimated statistic. We decided to perform interpolation based on spline function. Interpolation based on splines proved to be a better choice for phenomena which combine a random component as well as processes which minimize energy, as it could be considered socio-demographic processes [30]. We chose the function “regularized spline with tension” implemented in the GRASS-method  v.surf.rst [31]. This method computes the continuous 3D layer (raster data) simulating a thin flexible plate passing through or close to the measured data points; it is the most general and accurate method available in GRASS [30].

In order to run  v.surf.rst, point-data layers are required. For each LU, we first specified its geometrical center (*centroid*) with a GRASS basic module. Statistic values were linked to the centroids. We obtained one point-data layer for each measured statistic. In case of Augsburg City, which contains 14 postal areas and covers a disproportionately large area, centroids of all postal areas were used. Computed values for Augsburg City data were assigned to all its 14 centroids. With this step, we smoothed spatial interpolation results in the area of Augsburg City and surroundings, while avoiding interpolation artifacts. We modeled genetic landscapes based on tuned values of  v.surf.rst  parameters. In order to be able to adequately execute the  v.surf.rst  procedure the *H*_*O*_ values computed using the raw data (*H*_*O* raw_) were transformed into percentage as follows:

(2) $$\begin{array}{c} H_{O\;\text{raw}}\cdot 100=H_{O}, \end{array}$$

where *H*_*O* raw_ ∈ [0,1], *H*_*O*_ ∈ [0,100].

Interpolation surfaces based on *H*_*O*_ values were created for the following data sets: *H*_*O*_(ALL), and *H*_*O*_(GER). Interestingly, since the KORA S4 genotypes conform a control population pool for genetic studies [7, 9], *H*_*O*_(ALL) landscape may be examined as a representative estimation of the spatial variation of genetic diversity of the extant population and *H*_*O*_(GER) landscape of the native fraction in the region of Augsburg.

#### 2.7.2. Spatial Autocorrelation

The presence of simple association between the variability of an attribute and the geographical space was tested by means of spatial autocorrelation. In this case, the null hypothesis is that the feature of interest is spatially distributed at random among other attributes within the study area. This analysis was based on the Moran's *I* tests. Spatial correlation measured with the test statistic Moran's *I* is inferential, which implies that results must be interpreted in dependence of the null hypothesis. For this analysis we used a Global Moran's *I* statistic, which means that we tested for spatial autocorrelation in the study area as a whole, assuming that the spatial process is the same everywhere.

Spatial autocorrelation of each of the genetic and socio-demographic variables defined in this study was tested with the R package* spdep* [32].

Moran's *I* tests were performed using the function implementations  moran.test  and  moran.mc. Accounting for normality deviation of the data, moran.test  was run under the specification of randomization assumption in computing the variance of the statistic. This test specification allows relaxing the simpler normality assumption by introducing a correction term based on the kurtosis of the inspected variable.

The second implementation, moran.mc, is a permutation-based test. With this implementation spatial autocorrelation is evaluated independently of normality and randomization assumptions. The function  moran.mc  uses a Monte Carlo test, based on a permutation bootstrap. Observed values are randomly assigned to areal entities, and the value of the observed Moran's *I* is computed nsim  times [33]. We set  nsim  =  10 000. These tests were run using a binary matrix of spatial weights (see Section 2.3.4).

Both implementations,   moran.test  and  moran.mc, were used to test for spatial autocorrelation in measures of genetic variation: (*H*_*O*_(ALL), *H*_*O*_(GER)), as well as on the socio-demographic variables: GER_P, MAIN_IMP, AGE25_39, AGE40_54, EA55_74, AGE_MEAN, EY8_11, EY_MEAN, and EDU_MEAN.

#### 2.7.3. Search of Best Predictors

Socio-demographic measures were inspected as predictors of the observed pattern of *H*_*O*_(GER) under the assumption that socio-demography would provide useful indication of spatial arrangement of recent migration processes, specially regional and national migration, which we assumed that it must have had a strong influence on fine-scale genetic variation. The contribution of socio-demographic factors to explain the observed spatial pattern was analyzed under the assumption of spatial dependence. Best-fit spatial autoregressive models (SAR) predicting heterozygosity (*H*_*m*_) were selected. A stepwise forward search was conducted using the function  spautolm  of the package R* spdep* [32]. The function  spautolm  computes a regression on the values from the other areas to estimate the spatial dependence of the residuals of the specified linear predictor. The spatial dependence is estimated with a maximum likelihood test, computing a spatial autocorrelation parameter, *λ*. The *p* value of the likelihood ratio test compares the model with no spatial autocorrelation (*λ* = 0) to the one which allows for it [33]. A binary matrix of spatial weights was used for this analysis (see Section 2.3.4).

Model selection was started with following parameters: GER_P, AGE25_39, AGE40_54, EA55_74, AGE_MEAN, EY8_11, EY_MEAN, and EDU_MEAN. In order to test if the spatial distribution of the major immigrant group (MAIN_IMG) improves model prediction, the influence of the parameter MAIN_IMP on the selected model was tested.

A set of models best fitting the data were selected according to the *p* values of the covariates (*p* value < 0.05).

#### 2.7.4. Evaluation of Model Accuracy

Finally, the goodness of fit of the selected SAR models was analyzed. In this step, the pixelwise divergence between predicted (*H*_*m*_) and observed values (*H*_*O*_) was quantified. Interpolation surfaces were created based on the predicted values (*H*_*m*_) by each selected model. The pixelwise divergence in absolute values of these interpolation surfaces from the *H*_*O*_(*GER*) landscape was used to compare prediction accuracy among the selected SAR models. In order to facilitate comparison among models, a standardized difference was computed. The standardization was performed based on the maximal range of pixel values (max⁡_rg_GER_) measured in the *H*_*O*_(*GER*) landscape. The parameter max⁡_rg_GER_ was computed as follows:

(3) $$\begin{array}{c} {\text{max\_rg}}_{\text{GER}}={z\text{max\_int}}_{\text{GER}}-{z\text{min\_int}}_{\text{GER}}, \end{array}$$

where *z*max_int_GER_ is the maximal value measured in the *H*_*O*_(GER) landscape and *z*min_int_GER_ the minimal value.

For each SAR model, a new elevation surface (raster layer) storing the respective pixelwise difference was created. Following pixelwise computation was performed with the GRASS basic module  r.mapcalc:

(4) $$\begin{array}{c} \left(\;\frac{\text{abs}\left[H_{O}\left(\text{GER}\right)-H\left(m_{n}\right)\right]}{{\text{max\_rg}}_{\text{GER}}}\;\right)100, \end{array}$$

where abs implies absolute value, *H*_*O*_(GER) refers to the pixel values of the *H*_*O*_(GER) landscape, *H*(*m*_*n*_) refers to the pixel values of the interpolated surface created on the bases of the predicted *H* values of the *m*_*n*_ SAR model, and the parameter max_rg_GER_ = 1.64 (*z*min_int = 43.36; *z*max_int = 45.00).

For each one of these elevation surfaces, spatial global statistics were computed. For this purpose, elevation surfaces were imported into the spatial R environment provided by the packages  sp,  rgdal,  spdep, and  spgrass6  (R Foundation for Statistical Computing, 2010, http://www.r-project.org/foundation/). Mean, standard deviation (sd), median, minimum (min), and maximal values (max) of the elevation surfaces were computed with the R function  summary(). These statistics were applied as global quantitative measures of prediction goodness of each selected SAR model and were used to select the model best fitting the data.

The model with the lowest global difference between observed and predicted *H* values was selected as the one best fitting the data. Based on this model, maps representing the spatial variation of predicted *H* values and the estimated divergence between observed and predicted *H* values were created. The former map represents the estimated variation in heterozygosity according to predictions obtained by the SAR model best fitting the data. The latter maps allows a visual estimation of the agreement between observed and predicted heterozygosity in each land unit as well as the estimated spatial variation of divergence in the total study area.

## 3. Results

Descriptive statistics of all measures, including mean, standard deviation, median, minimum value, and maximum value, are presented in Table 2.

### 3.1. Spatial Variation of Socio-Demographic Factors

**Figure 3:**
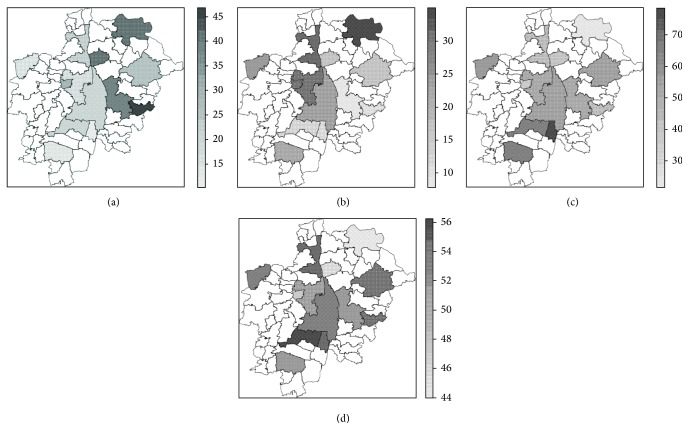
Values of age-related parameters; values refer only to German native subjects. (a) Percentage of subjects in the age category: 25–39 years (AGE25_39); (b) percentage of subjects in the age category: 40–54 years (AGE40_54); (c) percentage of subjects in the age category: 55–74 years (AGE55_74); (d) mean age per land unit (AGE_MEAN).

Age-related parameters showed a heterogeneous spatial distribution (Figure 3). Younger individuals (25 to 39 years old) comprised more than 30 percent of the GER sample in the eastern sector and reached a proportion of 45 percent in Pöttmes (Figure 3(a)). Subjects corresponding to the intermediate age category (40 to 54 years old) showed a lower proportion (less than 30 percent) in LUs contiguous to Augsburg City in the South and in the East (Figure 3(b)). The upper age category (55 to 74 years old) accounted for more than 50 percent in eight of the 13 LUs, presenting the higher proportions (>70%) southern from Augsburg City (Figure 3(c)). The lowest mean age values were recorded in Pöttmes, Rehling, and Neusäß (Figure 3(d)). The percentage of individuals of the intermediate and the upper age category (AGE40_54, AGE55_74) showed a deviation of the expected Moran's *I* value with both tests (moran.test,  moran.mc) on a significance level of *α* = 0.05. The percentage of individuals of the lower age category (AGE25_39) showed a significant deviation of the expected Moran's *I* value with  moran.test, but with the M-C permutation bootstrap test did not reach a significance at *α* = 0.05 (Table 3). The mean age (AGE_MEAN) did not show any indication of spatial dependence with either of both tests (Table 3).

**Figure 4:**
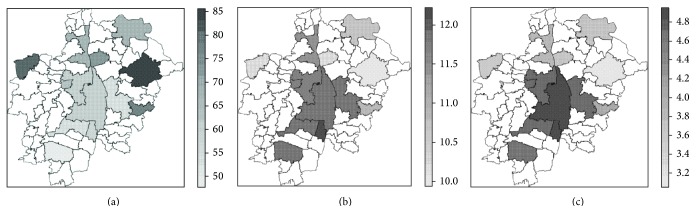
Values of education-related parameters; values refer only to German native subjects. (a) Percentage of subjects achieving a maximum of 11 school years, where the maximum in the sample is 17 school years (EY8_11); (b) mean years of school attendance per LU (EY_MEAN); (c) mean score of the education level per LU, ranging from 0 = no school degree up to 9 = graduate degree (M.S. equivalent or higher).

With regard to the spatial distribution of education level in the study area, the largest values of education years, that is, lower values of EY8_11, were observed in Augsburg City and in neighboring LUs in the East (Friedberg) and in the West (Aystetten, Neusäß), as well as in the southern LU of Schwabmünchen (Figure 4(a)). Both mean variables, means of education years and education level (EY_MEAN, EDU_MEAN), showed the largest values in the center and in the South of the study area, while the peripheral LUs Aichach and Altenmünster showed the lowest values (Figures 4(b) and 4(c)). Whereas education level (EDU_MEAN) showed a significant deviation from the expected Moran's *I* value with both tests (moran.test,  moran.mc) at *α* = 0.05, the education years (EY_MEAN) presented just an indication of potential spatial dependency at this significance level (Table 3).

**Figure 5:**
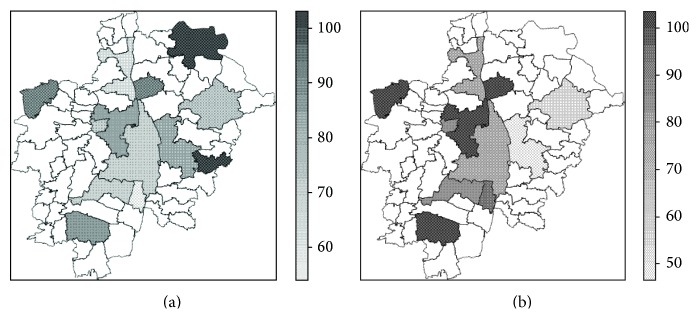
Spatial distribution of samples according to land of birth. (a) Spatial distribution of GER_P; (b) spatial distribution of MAIN_IMP.

The percentage of natives (GER_P) showed a complex pattern (Figure 5(a)). Considering LUs with an intermediate number of samples, the percentage of natives decreased with an increase of the absolute number of samples per land unit with the exception of Altenmünster and Neusäß (Table 1). In Augsburg City, with a quite larger number of samples in comparison with all other LUs (Table 1), natives composed ca. 70 percent of the total samples (Figure 5(a)). The lowest percentages were measured around Augsburg City, in Königsbrunn, followed by Langweid. Bobingen, contiguous to Augsburg City on the South, and Aystetten, relatively peripheral to Augsburg City, presented the next lower frequencies of natives (Figure 5(a)). Samples included only natives in the eastern peripheral LUs, Pöttmes and Eurasburg (Figure 5(a)), both accounting for the lowest sample counts as well (Table 1). The distribution of the proportion of natives (GER_P) did not show any indication of a potential spatial dependency (Table 3).

The major group of immigrants (MAIN_IMG) showed a higher ratio in the western LUs (Figure 5(b)). More than half of the units showed values of MAIN_IMP larger than 80 percent. In Augsburg City, where a considerably larger total number of immigrants were sampled, the major immigrant group composed 78 percent of the total immigrant data set (Table 1). The results of both Moran's *I* tests of spatial autocorrelation (moran.test,   moran.mc) pointed to a potential simple spatial dependency of the parameter MAIN_IMP, which however did not reach a significance level of *α* = 0.05 (Table 3).

### 3.2. Geographic Variation of Genetic Diversity

The search for indications of spatial patterns of genetic heterogeneity with well-established procedures did not provide any positive results. Although a high number of different explorative runs with different parameters were performed, tests based either on EIGENSTRAT | EIGENSOFT, PLINK, GENELAND, or SPAGeDI did not provide any indication of a potential geographic variation of genetic heterogeneity in the study area. A brief summary of a representative extract of these computations is presented in Supplement 5.

Geostatistical analysis based on the statistic observed heterozygosity (*H*_*O*_) [22] provided indication of spatial patterning. Table 3 presents results of the spatial autocorrelation analysis performed with test statistic Moran's *I*. On the one side, the variable *H*_*O*_(ALL) showed an indication of association between genetic variation and geographic coordinates. On the other, in the native sample, tests of global spatial autocorrelation showed a significant deviation (on a significance level of *α* = 0.05) of a random spatial distribution of *H*_*O*_(GER) values (Table 3). This result was obtained with both function implementations  moran.test  and  moran.mc. Significant results obtained in the native sample may indicate that the additional genetic variability contributed by the immigrant fraction of the sample could introduce noise, which diluted a subtle patterning of the genetic attributes of the native sample.

**Figure 6:**
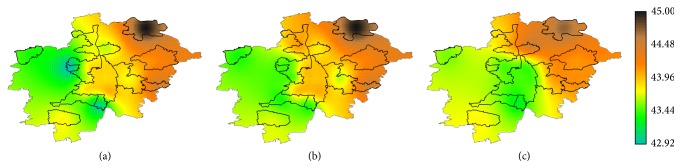
Landscapes estimating the genetic variation in the study area (a) *H*_*O*_(ALL): observed heterozygosity of the total sample (officially registered German citizens); (b) *H*_*O*_(GER) observed heterozygosity of the native data set (individuals born in Germany); (c) predicted heterozygosity according to the best-fit spatial autoregressive model (Table 4).

*H*_*O*_(ALL) landscape, which estimates the variation of the genetic heterogeneity of the extant German population in the study area, presented a marked depression in the East (Figure 6(a)). The highest *H*_*O*_ values were measured in the eastern area. Intermediate *H*_*O*_ values covered the central-northern sectors, including some areas of Aichach-Friedberg District, Langweid, Augsburg City, and Schwabmünchen. The lowest *H*_*O*_ values were observed in the western area. The minimum values were found in Neusäß and Königsbrunn.

The *H*_*O*_(GER) landscape, estimating the spatial variation of genetic heterogeneity of the native population, showed similar values to the *H*_*O*_(ALL) landscape in the western and in the eastern periphery. In the central belt, running across the study area in north-south direction, this landscape showed higher values than the *H*_*O*_(ALL) landscape (Figure 6(b)).

**Figure 7:**
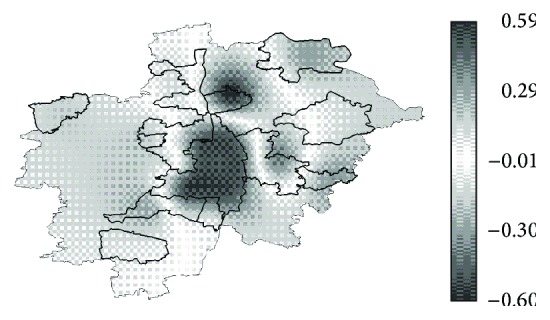
Difference between the interpolated surfaces of the observed and the predicted heterozygosity of the native subset (*H*_*O*_(GER) − *H*(*m*_2_)).

Four spatial autoregressive models were selected according to the *p* value (*p* value < 0.05) of the covariates (Table 4). The four models included as covariates variables related to age and education; two models (*m*_1_, *m*_3_) included as well the proportion of natives per LU (GER_P), which is at the same time an indication of the proportion of immigrants per LU. The inclusion of the variable MAIN_IMP, which involves a differentiation of subgroups of immigrants, did not improve any of the selected models. Out of these four selected models *m*_2_ showed a significant *p* value when the likelihood ratio of *λ* was tested, indicating left spatial correlation in the residuals (*p* value = 0.018). The *H*(*m*_2_) landscape (Figure 6(c)) showed as well the lowest mean and standard deviation of pixelwise difference to the *H*_*O*_(GER) landscape (Table 4), which we used as indicators of model goodness. The pixelwise difference between *H*_*O*_(GER) and *H*(*m*_2_) surfaces showed a good agreement over a large area as well. Areas where both surfaces showed very similar values are indicated in Figure 7 with a white or light grey and correspond to a pixelwise *H* difference close to zero. The maximal differences were measured in Augsburg City and in Rehling, a small residential area in the country side. Best fitting was obtained in the peripheral ring surrounding Augsburg City (Figure 7).

## 4. Discussion and Conclusions

Fine-scale variation of genetic heterogeneity within a small region was detected and analyzed applying a geographic perspective. Population genetics and geostatistics were combined with the open-source geographic information system GRASS. The capabilities of this approach were tested on a subset of the KORA S4 survey [7, 9], collected in southern Germany for prospective studies. Tests on this data set with the well-known software STRUCTURE previously reported by others [14] did not provide evidence for population substructure. We assumed that within small urbanized areas of modern western countries, as is the case in Germany, genetic composition may be strongly affected by migratory movements of the last half century, which may be still estimated by means of socio-demographic measures.

Genotypes (212 autosomal SNPs) and socio-demographic information (age, education, place of residence, and birth land) of 728 healthy German citizens were analyzed.

Socio-demographic and genetic measures showed heterogeneous distribution across the study area. The estimated values of the observed heterozygosity showed to some extent a cline of decreasing values from east to west. In a first step to analyze spatial processes controlling the observed patterns, Moran's *I* tests for spatial autocorrelation were performed for all available parameters. Indications of global spatial dependencies were observed in socio-demographic variables related to age categories and education level. While significant deviation of the expected Moran's *I* was obtained with the observed heterozygosity of the native subset (GER), the observed heterozygosity of the total data set showed just an indication of a potential deviation of the expected Moran's *I*. In other words, these results could be interpreted as an indication that heterozygosity values of this small area may be regulated by a global spatial process, but such subtle process could only be observed when the subset of native Germans is considered. This is consistent with the elemental assumption that because immigrants may differ to some extent in their genetic background, and this would be reflected in their observed heterozygosity, they would introduce additional variability which hinders detection of spatial variability pattern of the most frequent group, the German natives.

Spatial dependencies of socio-demographic variables at this local level could be axiomatically interpreted as a result of neighborhood preferences of recent local and regional migratory processes. It is to expect that social preferences affecting recent migration, such as regional and international migratory movements of the last half century, may be reflected by socio-demographic parameters. Individuals would not choose new residence randomly and this may be reflected in similar socio-demographic attributes of contiguous neighborhoods. This expectation, which goes along with common sense, would also be in agreement with the fact that the level of admixture would associate to some extension with socio-demographic attributes of the location. This would be the result of new and recurrent resettlement of some areas or that inhabitants of areas presenting certain socio-demographic features would show higher predisposition to admixture. Indeed, the fact that patterns of socio-demographic structure and genetic admixture account for spatial autocorrelation could indicate the occurrence of further unobserved phenomena influencing both, as it could be “social preferences” or “official urban planning.” Nevertheless, it went beyond the scope of this work to search for such unobserved processes.

Spatial autoregressive models (SAR) fitting the data were selected by a forward search. The four best-fit SAR models contained as covariates socio-demographic measures related to age, school-attendance years, and education level. Two of the four selected models included as well the percentage of natives per unit. This parameter directly accounts for degree of demographic admixture in sense of proportion of immigrants as measured by the parameter “land of birth.” The prediction strength of the selected model was estimated with quantitative comparisons between the genetic landscapes created on basis of observed and predicted measures of heterozygosity. We found a good agreement between the predicted and the observed patterns, which supports the assumption of a certain relationship between genetic admixture degree and socio-demographic structure. Predicted and observed values showed the highest agreement in the surrounding belt of Augsburg City. The largest deviations were measured in Augsburg City itself and in two small residential areas in the eastern countryside. The differentiation of Augsburg City to the countryside (see Supplement 3) may be interpreted as the expected divergence of middle-size industrial settlements and countryside [34]. This effect could still be detected even after excluding immigrants, subjects born outside of Germany. This expected differentiation between urban and countryside areas in relation to genetic heterozygosity may be considered as a confirmation that the observed patterns are not artifacts.

The differentiated area in the countryside, Rehling, corresponded to a land unit with relatively low number of samples. The lower predictive capability of the best-fit model could be attributed to sampling bias. A replication would be necessary to verify this conjecture. A further possibility could be that this settlement offers an attractive residential area for individuals working in any of the larger urban centers located eastern from Augsburg, as it could be Ingolstadt or Munich. Both urban centers offer highly profitable working alternatives, in sense of carrier opportunities and higher income, and act as an attraction pole for domestic and foreign migration. As well, both cities are also among the ones with the most expensive living costs. Rehling showed also the largest proportion of younger adults. This may reinforce the idea that this location may be attractive for newly settled workers willing to commute between their working place and a less expensive residential area, relatively close to a middle-size urban center as Augsburg. If this would be the case, the inclusion of distance and accessibility to attractive urban centers could considerably improve model prediction. Further studies could test this possibility.

The exclusion of immigrants increased the global mean of observed heterozygosity. This effect was especially strong in areas accounting for the largest proportion of immigrants. About 15 percent of the samples were immigrants, born worldwide. Remarkably, almost 80 percent of them corresponded to individuals born either in Czech Republic, Romania, Poland, or Ukraine. Some areas situated in the periphery of Augsburg City showed a stronger component of immigrants, mostly or totally represented by this major immigrant group. Although this group involved four birth lands, it may be speculated that the concentration of these individuals is not casual. It could be expected that, within each provenance group, individuals could be originals of nearby regions or belonging to large related families. In total, a concentration of small groups, each one showing a higher degree of homozygosity, would stand out over a much more admixed group, as it is expected for the native German population.

Our additional analysis with vastly cited multivariate methods, GENELAND, EIGENSTRAT | EIGENSOFT, PLINK, and SPAGeDI, did not also submit indication of population stratification in the study area. This outcome is consistent with our expectations. These tools proved to be successful in studies of groups with considerably larger genetic differences, significantly more polymorphic loci, or much larger number of loci than in the present work. The KORA study was carefully designed for prospective studies aiming to reduce any type of genetic structuring. Following this objective, only German citizens were included in the sample. The sampling area was kept very circumscribed as well. Consequently, the genetic differentiation of this subset of the KORA cohort is expected to be considerably lower than in humans studies conducted at broader geographical scales or in further studies acknowledging larger evolutionary histories and dimensions (i.e., nonhuman species or samples with large population differences). A further aspect to be considered is the population informativeness of the available SNPs. These loci [14] were not specifically selected by their informativeness for distinguishing among major regional groups (i.e., [35, 36]). For this reason, the number of available SNPs was probably too low for detecting fine-scale population differences with these standard tools.

Using full capabilities of tools such as SPAGeDI or GENELAND, both offering individual-based analysis tools, was not possible either since the search of patterns of variation of genetic heterogeneity of the KORA sample cannot be carried out on an individualized geographical basis.

Data of human genetic studies would most probably not include an individualized geographical reference. Official restrictions concerning personal anonymity forbid the use of data which could individualize a subject, such as postal address or any other precise geographical reference. Therefore, for human studies, precision of geographic references must be kept low with the consequence that data might be spatially aggregated. In opposite to studies analyzing other species with a continuous geographical distribution, the identification of spatial structures of humans inhabiting small areas may not be carried out on an individual basis. Such an approach would jeopardize personal anonymity and would go against most official restrictions of human studies. Accordingly, it is not surprising that well-established tools to detect human population stratification on a broader level or those which make use of geographic references on an individual basis (mostly developed for studies of other species) may not detect fine-scale patterns of genetic variation in small areas.

Most genetic studies on modern human groups address recruitment of control samples on extant populations or make use of available control cohorts. These samples may present some degree of heterogeneity even if recruitment was restricted to small areas, by citizenship, or in combination with homogeneous place of birth. The degree of spatial heterogeneity of small geographic areas, frequently assumed to be neglectful in the context of genetic studies of modern human groups, should be evaluated on a case-by-case basis. Based on our outcomes, it could be stated that genetic heterogeneity could not be automatically assumed to be negligible. These results support the elemental assumption that within multiethnic, urban, and suburban groups, as found in medium-sized German cities and surroundings, the socio-economic parameter “birth land” allows a first reduction of genetic heterogeneity.

As it was presented in this study (see Supplement 2), even after removing immigrants from the KORA S4 data set, the degree of genetic differentiation in natives still overlapped with the spatial frequency distribution of immigrants. If future studies verify our exploratory results obtained with visual examination of genetic landscapes based on *F*_ST_ analogous Reynolds' *R* genetic distance, potentially, the proportion of immigrants may be used as subrogate of degree of natives' admixture, which could actually reflect native's behavior in regard to choice of area of residence and tolerance or predisposition to admix.

Our results about a differentiation among urban, suburban, and periurban population are a suggestion of a true effect in the sense of subtle population differentiation (cf. [34]). As mentioned above in this section, it is to assume that such differentiation would not result from isolation between neighboring areas in the sense of evolutionary processes, but from differences in regard to the degree of migration, residential preferences and willingness to intermix.

However, it must be stressed that our analysis is preliminary and it is predominantly aimed at a methodological evaluation. In particular, the coverage of the area is patchy and far from complete. In order to make use of these results for further genetic studies, first, the postulated fine-scale variation of genetic heterogeneity should be confirmed with a larger data set. Second, the magnitude of the detected bias for the corresponding analysis should be evaluated. A comprehensive analysis on an augmented data set is in preparation.

Knowledge of fine-scale patterns of genetic variation could provide information about areas where expected genetic heterogeneity could introduce undesired bias. Areas with an observed higher genetic heterogeneity than tolerable could be avoided. In case that spatial heterogeneity would be assessed after recruitment, examining the spatial pattern of genetic heterogeneity could serve as a basis to decide about a stratified analysis (e.g., grouping samples according to residence or any other relevant spatial reference) or to correct for population stratification (cf. [26, 27]). Our vision is to further develop our approach in order to be capable of testing as well as detecting and correcting, if it is applicable, for spatial patterns of genetic heterogeneity within the study sample (cf. [26, 27]). In contrast to the method implemented in EIGENSOFT | EIGENSTRAT [26, 27], which infers strata based on genetic data alone, such approach would make use of information on subject area membership to define the strata. This usage of additional* a priori* information potentially leads to improve strata definition (cf. [37]).

Taking these findings of the KORA S4 sample altogether we can state that fine-scale spatial genetic variation may be assumed in the study area. Our results indicate that patterns of genetic heterogeneity can be present in small regions within Germany. In conclusion, it may be stated that the presented genetic geostatistical approach has the potential of being a powerful tool for detecting, modeling, and analyzing spatial patterns of genetic heterogeneity even within populations inhabiting small regions.

## Supplementary Material

Supplement 1 describes the KORA S4 sample.
Supplement 2 presents additional analysis of spatial genetic differentiation based on conventional measures.
Supplement 3 presents an analysis of genetic differentiation between urban and peri-urban areas.
Supplement 4 provides a sensitive analysis of the effect on genetic variation of immigrants in the KORAS4 Survey.
Supplement 5 summarizes results of additional multivariate analyses of spatial genetic differentiation.
Supplement 6 describes the KORA S4 marker set.
Supplement 7 provides an analysis of genetic relatedness of individuals included in the KORAS4 sample.

## Figures and Tables

**Table 1:** Description of LUs and sampling locations, total count of samples, natives, immigrants, MAIN_IMG (subset of German citizens born in either Czech Republic, Romania, Poland, or Ukraine), and values of MAIN_IMP (percentage of individuals out of the total count of immigrants per land unit corresponding to the major immigrant group). Sampling locations with a low number of samples, which were aggregated to a contiguous sampled unit, are indicated in square brackets.

LU-ID	LU/sampling location	All (*n*)	Natives (*n*)	Immigrants (*n*)	MAIN_IMG (*n*)	MAIN_IMP (%)
1	Augsburg	359	258	101	79	78
2	Aichach	23	18	5	3	60
3	Eurasburg	9	9	0	0	0
4	Friedberg	25	21	4	2	50
5	Pöttmes	12	12	0	0	0
6	Rehling	13	12	1	1	100
7	Bobingen	51	36	15	13	87
8	Königsbrunn	42	24	18	17	94
9	Langweid	34	22	12	9	75
	[Meitingen]					
10	Neusäß	60	52	8	8	100
	[Gersthofen]					
	[Stadtbergen]					
11	Altenmünster	53	48	5	5	100
12	Aystetten	31	23	8	7	88
13	Schwabmünchen	16	14	2	2	100

**Table 2:** Descriptive statistics of genetic diversity and socio-demographic measures per LU (mean, standard deviation, median, minimum value, and maximum value).

Variable	Mean	SD	Median	Min	Max
*H*_*O*_ (ALL)	43.76	0.54	43.70	42.96	45.02
*H*_*O*_ (GER)	43.84	0.49	43.68	43.36	45.02
GER_P	81.5	13.2	84.0	57.0	100.0
MAIN_IMP	71.7	35.5	87.0	0.0	100.0
AGE25_39^1^	26.9	11.3	22.7	12.5	44.4
AGE40_54^1^	21.3	8.5	21.4	9.5	33.3
AGE55_74^1^	51.8	13.1	52.4	25.0	75.0
AGE_MEAN^1^	52.0	3.6	53.2	44.8	55.5
EY8_11^1^	66.0	10.6	65.2	50.0	83.3
EY_MEAN^1^	11.3	0.7	11.7	10.1	12.1
EDU_MEAN^1^	4.3	0.6	4.6	3.2	4.8

^1^Age and education-related variables refer only to the native group (GER).

**Table 3:** Estimated Moran's *I* values and *p* values of two Moran's *I* tests for spatial autocorrelation computed for all defined variables.

Measure	Moran's *I *	*p* value^1^	*p* value (MC)^2^
*H*_*O*_ (ALL)	0.1132	*0.074 *	*0.090 *
*H*_*O*_ (GER)	0.2095	**0.015**	**0.024**
AGE25_39	0.1823	**0.039**	0.054
AGE40_54	0.3220	**0.004**	**0.010**
AGE55_74	0.2326	**0.013**	**0.020**
AGE_MEAN	−0.1092	0.571	0.550
EY8_11	0.0624	0.164	0.159
EY_MEAN	0.1425	*0.066 *	*0.075 *
EDU_MEAN	0.2509	**0.013**	**0.029**
GER_P	−0.1945	0.775	0.762
MAIN_IMP	0.1194	*0.072 *	*0.091 *

^1^Computed using the R package **spdep** m oran.test  based on a randomisation assumption.

^2^Computed using the R package **spdep** moran.mc, consisting of a Monte Carlo test, based on a permutation bootstrap test; *p* values were obtained on 10 000 runs.

**Table 4:** Best spatial autoregressive models fitting the data. The spatial autocorrelation left in the residuals (*λ*) and the *p* value of the likelihood ratio test, comparing the residuals of the fitted model with the one with no spatial autocorrelation (i.e., *λ* = 0), are indicated for each model. In order to compare these four models, landscapes were created as the pixelwise difference between the observed and the predicted genetic landscape for each *n* model (*H*_*O*_ (GER) − *H* (*m*_*n*_)). Differences were computed in percentage to the maximal range of values of the *H*_*O*_ (GER) landscape. Mean, standard deviation (SD), and maximal values (Max) of the differences are indicated.

Model		*λ*	*p* value	Mean	SD	Max
*m*_1_	40.768 + GER_P (0.014) + AGE40_54 (0.031) + EY8_11 (0.021)	0.203	0.449	17.0	14.9	70.6

*m*_2_	48.903 + AGE25_39 (0.028) + EY8_11 (−0.036) + BILD_MN (−0.802)	−0.309	*0.018 *	10.0	7.6	36.6

*m*_3_	38.897 + GER_P (0.014) + EA55_74 (−0.013) + EY_MN (0.881) + BILD_MN (−1.293)	0.130	0.590	13.2	8.4	38.4

*m*_4_	52.457 + EA55_74 (−0.031) + AGE_MN (0.090) + EY_MN (−0.811) + EY8_11 (−0.038)	−0.159	0.451	11.8	8.2	39.9

## Abbreviations

GIS: Geographic Information System
SNP: Single Nucleotide Polymorphism
MC: Monte Carlo.

## References

[1] Bamshad M. J., Wooding S., Watkins W. S., Ostler C. T., Batzer M. A., Jorde L. B. (2003). Human population genetic structure and inference of group membership. *American Journal of Human Genetics*.

[2] Cardon L. R., Palmer L. J. (2003). Population stratification and spurious allelic association. *The Lancet*.

[3] Sloan C. D., Duell E. J., Shi X. (2009). Ecogeographic genetic epidemiology. *Genetic Epidemiology*.

[4] Freedman M. L., Reich D., Penney K. L. (2004). Assessing the impact of population stratification on genetic association studies. *Nature Genetics*.

[5] Marchini J., Cardon L. R., Phillips M. S., Donnelly P. (2004). The effects of human population structure on large genetic association studies. *Nature Genetics*.

[6] Shriver M. D., Kennedy G. C., Parra E. J. (2004). The genomic distribution of population substructure in four populations using 8,525 autosomal SNPs. *Human genomics*.

[7] Holle R., Happich M., Löwel H., Wichmann H. E. (2005). KORA—a research platform for population based health research. *Gesundheitswesen*.

[8] Lindenberg A., Brinkmeyer J., Dahmen N. (2011). The German multi-centre study on smoking-related behavior-description of a population-based case-control study. *Addiction Biology*.

[9] Wichmann H.-E., Gieger C., Illig T. (2005). KORA-gen—resource for population genetics, controls and a broad spectrum of disease phenotypes. *Gesundheitswesen*.

[10] Helgason A., Yngvadòttir B., Hrafnkelsson B., Gulcher J., Stefánsson K. (2005). An Icelandic example of the impact of population structure on association studies. *Nature Genetics*.

[11] Epperson B. K. (2003). *Geographical Genetics*.

[12] Kaessmann H., Wiebe V., Weiss G., Pääbo S. (2001). Great ape DNA sequences reveal a reduced diversity and an expansion in humans. *Nature Genetics*.

[13] Laland K. N., Odling-Smee J., Myles S. (2010). How culture shaped the human genome: bringing genetics and the human sciences together. *Nature Reviews Genetics*.

[14] Steffens M., Lamina C., Illig T. (2006). SNP-based analysis of genetic substructure in the German population. *Human Heredity*.

[15] Wright S. (1969). *Evolution and the Genetics of Populations, Vol. 2: Theory of Gene Frequencies*.

[16] Pritchard J. K., Stephens M., Donnelly P. (2000). Inference of population structure using multilocus genotype data. *Genetics*.

[17] Wright S. (1951). The genetical structure of populations. *Annals of Eugenics*.

[18] Abecasis G. R., Cherny S. S., Cookson W. O. C., Cardon L. R. (2001). GRR: graphical representation of relationship errors. *Bioinformatics*.

[19] Devlin B., Roeder K. (1999). Genomic control for association studies. *Biometrics*.

[20] Guillot G., Estoup A., Mortier F., Cosson J. F. (2005). A spatial statistical model for landscape genetics. *Genetics*.

[21] Nei M., Roychoudhury A. K. (1974). Sampling variances of heterozygosity and genetic distance. *Genetics*.

[22] Nei M. (1973). Analysis of gene diversity in subdivided populations. *Proceedings of the National Academy of Sciences*.

[23] Wright S. (1943). Isolation by distance. *Genetics*.

[24] Wright S. (1946). Isolation by distance under diverse systems of mating. *Genetics*.

[25] Guillot G., Santos F., Estoup A. (2008). Analysing georeferenced population genetics data with Geneland: a new algorithm to deal with null alleles and a friendly graphical user interface. *Bioinformatics*.

[26] Patterson N., Price A. L., Reich D. (2006). Population structure and eigenanalysis. *PLoS Genetics*.

[27] Price A. L., Patterson N. J., Plenge R. M., Weinblatt M. E., Shadick N. A., Reich D. (2006). Principal components analysis corrects for stratification in genome-wide association studies. *Nature Genetics*.

[28] Purcell S., Neale B., Todd-Brown K. (2007). PLINK: a tool set for whole-genome association and population-based linkage analyses. *American Journal of Human Genetics*.

[29] Hardy O. J., Vekemans X. (2002). SPAGeDi: a versatile computer program to analyse spatial genetic structure at the individual or population levels. *Molecular Ecology Notes*.

[30] Neteler M., Mitasova H. (2004). *Open Source GIS: A GRASS GIS Approach*.

[31] Mitášová H., Mitáš L. (1993). Interpolation by regularized spline with tension: I. Theory and implementation. *Mathematical Geology*.

[32] Bivand R. http://cran.r-project.org/web/packages/spdep/index.html.

[33] Bivand R. S., Pebesma E. J., Gomez-Rubio V. (2008). *Applied Spatial Data Analysis with R*.

[34] Vitart V., Carothers A. D., Hayward C. (2005). Increased level of linkage disequilibrium in rural compared with urban communities: a factor to consider in association-study design. *American Journal of Human Genetics*.

[35] Liu N., Chen L., Wang S., Oh C., Zhao H. (2005). Comparison of single-nucleotide polymorphisms and microsatellites in inference of population structure. *BMC Genetics*.

[36] Rosenberg N. A., Li L. M., Ward R., Pritchard J. K. (2003). Informativeness of genetic markers for inference of ancestry. *American Journal of Human Genetics*.

[37] Manel S., Berthoud F., Bellemain E. (2007). A new individual-based spatial approach for identifying genetic discontinuities in natural populations. *Molecular Ecology*.

