# Supplementary material for: Genetic Geostatistical Framework for Spatial Analysis of Fine-Scale Genetic Heterogeneity in Modern Populations: Results from the KORA Study
Source: Int J Genomics. 2015 Jul 16;2015:693193. doi: 10.1155/2015/693193 (PMC4519554; doi:10.1155/2015/693193)
Supplement: Supplementary file 1 — Supplement 1 describes the KORA S4 sample. Supplement 2 presents additional analysis of spatial genetic differentiation based on conventional measures. Supplement 3 presents an analysis of genetic differentiation between urban and peri-urban areas. Supplement 4 provides a sensitive analysis of the effect on genetic variation of immigrants in the KORAS4 Survey. Supplement 5 summarizes results of additional multivariate analyses of spatial genetic differentiation. Supplement 6 describes the KORA S4 marker set. Supplement 7 provides an analysis of genetic relatedness of individuals included in the KORAS4 sample. [file 693193.f1.pdf]

Supplementary Material

Genetic Geostatistical Framework for Spatial Analysis of Fine-Scale Genetic Heterogeneity in Modern Populations

# - Results from the KORA Study

Diaz-Lacava AN[1,6,7], Walier M[1], Holler D[1], Steffens M[1], Gieger C[3,9], Furlanello C[5], Lamina C[8], Wichmann HE[4,10,11], Becker T[1,2]

Institution

1: Institute for Medical Biometry, Informatics, and Epidemiology, University of Bonn, Bonn 53127, Germany

2: German Center for Neurodegenerative Diseases, DZNE, Bonn 53127, Germany

3: Research Unit of Molecular Epidemiology, Helmholtz Zentrum München, German Research Center for Environmental Health, Neuherberg 85764, Germany.

4: Institute of Medical Informatics, Biometry and Epidemiology, Ludwig-Maximilians-University, Munich 81377, Germany.

5: FBK, Trento 38122, Italy

6: Cologne Center for Genomics, University of Cologne, Cologne 50931, Germany

7: DNA Analysis Unit, Official College of Pharmacists and Biochemists, Buenos Aires C1184ABA, Argentina

8: Division of Genetic Epidemiology, Department of Medical Genetics, Molecular and Clinical Pharmacology, Medical University of Innsbruck, Innsbruck 6020, Austria

9: Institute of Epidemiology II, Helmholtz Zentrum München, German Research Center for Environmental Health, Neuherberg 85764, Germany.

10: Institute of Epidemiology I, Helmholtz Zentrum München, German Research Center for Environmental Health, Neuherberg 85764, Germany.

11: Institute of Medical Statistics and Epidemiology, Technical University Munich, Munich 81675, Germany.

Corresponding Author

Amalia Diaz-Lacava
diaz-lacava@imbie.meb.uni-bonn.de

Keywords: genetic heterogeneity, GIS, geostatistics, genetic landscape, social-demography

Running head: GIS-Assessment of fine-scale Genetic Heterogeneity

Nonstandard abbreviations: GIS: geographic information system; SNP: single nucleotide polymorphism; MC: Monte Carlo.

Supplement 1

KORA S4 Sample

Parameter	Entire sample<br>*n* = 728	Natives<br>*n* = 549	Immigrants<br>*n* = 179	Main Immigrant<br>*n* = 146
Age in years (SD)	54.3<br>(13.2)	52.7<br>(13.6)	59.3<br>(10.8)	59.6<br>(10.5)
% Females	49.6	51.7	43.0	42.5
Average years of school attendance (SD)	11.5<br>(2.6)	11.6<br>(2.7)	11.0<br>(2.4)	11.1<br>(2.3)
% College or advances studies	27.5	28.4	24.6	25.3

Supplementary Table S1 Social-demographic variables of the KORA S4 sample. This cohort included only German citizens. According to the place of birth, individuals can be divided into *natives* (individuals born in Germany), *immigrants* (individuals born outside of Germany) and the *main group of immigrants* (subset of German citizens born either in: Czech Republic, Romania, Poland, or Ukraine).

Supplement 2

Conventional Measures of Spatial Genetic Differentiation

Materials and methods

Contingency Tables

In a first explorative step, a traditional test for detecting spatial stratification of allele frequencies, χ^2 test for contingency tables (Weir 1996), was conducted. This test evaluates the independence of a feature variation and the units of analysis. If applicable to spatial units, this can be considered a test of spatial independence of the variability of the specific attribute.

This analysis included data from all 16 settlements, each one representing in this evaluation a land unit of analysis (LU) (see Land Units). For each SNP a 16x2 contingency table of allele counts was defined, where the rows represent the respective LU and the two columns represent the two alleles (data set: ALL). Only those autosomal markers where both alleles were present in all LUs were tested (182 SNPs; 64 intragenic SNPs; 118 intergenic SNPs; see Supplementary Table S6). Allele counts, instead of genotype counts, were considered for two reasons. Firstly, they greatly reduce the number of degrees of freedom that have to be considered. Secondly, gene flow (a result of migration and admixture), is assumed to be the major process affecting the genetic structure of modern admixed populations inhabiting small areas. The effects of gene flow are expected to be adequately represented in differences of allelic frequencies. Consequently, the use of genotypes would unnecessarily increase computation complexity.

For each marker, we considered the null hypothesis (H_0) that the allele frequencies are equal in all LUs. In a screening step, asymptotic p values for the respective contingency tables were computed, using the χ^2 distribution (Weir 1996). In order to overcome artifacts caused by small cell counts, we confirmed the ten best p values using a permutation test based on Monte-Carlo (MC) simulations. Although LUs contain different sample numbers, the test we applied is a valid test since we used an MC-simulation strategy.

The analysis was based on FAMHAP (Becker and Knapp 2004). FAMHAP is a program for single-marker and haplotype association analysis. In particular, it implements a permutation test for case-control data that is based on MC simulations. This method was used here to account for small cell counts. Several steps were taken. First, an analysis was performed with the “hapcc” method. This method was originally designed to obtain a permutational analogue of the χ^2 test for contingency tables whose rows refer to alleles (or haplotypes) and whose columns refer to case/control status (Becker et al. 2005). The resulting dataset was recoded in the computational table as detailed below. For the following arguments, let a_i be the total count of allele 1 in LU *i*, and let b_i be the count of allele 2 in LU *i*, [$1 \leq i \leq 16$]. We then considered a data file with 16 pseudo alleles, corresponding to the LUs, and a pseudo case-control status, corresponding to the two true SNP alleles, respectively. For each LU *i*, we added a_i “affected” individuals who were homozygous for pseudoallele *i* and b_i “unaffected” individuals who were homozygous for a pseudoallele *i* to a data file. The evaluation of this data file with FAMHAP and the “hapcc” option then yields a permutational analogue of the χ^2 test for our original contingency table. Note that the homozygous coding is naturally accounted for by the permutation procedure described in (Becker et al. 2005). Finally, the coding scheme made it possible to also use the “hapccmax” option of FAMHAP [http://www.uni-bonn.de/~umt70e/becker.html]. While accounting for the number of rows considered, this method considers each row (= allele or LU) with the most extreme cell count distribution rather than on the whole distribution. As it was not possible with this data set to discern between real and random effects, this line of evaluation was no further followed.

Computation of Genetic Distances

Spatial patterns of genetic heterogeneity were examined with a well-stablished population measure of genetic distance: Reynolds’ D_R genetic distance (Reynolds et al. 1983). Reynolds’ D_R genetic distance is a Wright’s F_{ST} analogous measure. It was specifically proposed for short-term genetic distance between groups when mutation accumulated in evolutionary time scales can be neglected (Reynolds et al. 1983). Reynolds’ genetic distance was computed using $D_R = -ln[1 - F_{ST}]$ (Reynolds et al. 1983), where F_{ST} is the heterozygote deficiency due to population subdivision (Wright 1951).

Reynolds’ D_R was computed with the module “dist.genet” of the R-statistics package ade4 [http://pbil.univ-lyon1.fr/ADE-4]. To avoid outlier bias introduced by units with low number

of observations of the whole data set (ALL) distributed in 16 LU (ALL/LU16), genetic-heterogeneity measures were tested on the resample set of 13 LU (see Land Units), both in the total data set as well as in the subset of natives (data sets ALL/LU13 and GER/LU13 respectively).

Results

Spatial Comparison of Allele Frequencies

Assuming the null hypothesis, i.e. that there is no association between allele frequencies and the geographical space, the screening step yielded 10 SNPs which were significant at an α level of 0.05, tested with a χ^2 test on contingency tables.

.

MARKER	χ^2	hapccmax	hapcc	location (gene)
rs597354	0.0026	0.0378	0.0046	intergenic
rs461311	0.0059	0.0684	0.0058	intergenic
rs717477	0.0085	0.0016	0.0068	intergenic
rs2242046	0.0095	0.0467	0.0102	intragenic
rs1860300	0.0161	0.0022	0.0141	intergenic
rs3625	0.0336	0.0059	0.0414	intragenic
rs896664	0.0419	0.0693	0.0549	intergenic
rs1997660	0.0437	0.1032	0.0253	intragenic
rs4379869	0.0445	0.0975	0.0566	intragenic
rs927470	0.0494	0.1488	0.0560	intergenic

Supplementary Table S2-1 List of markers with *p* values < 0.05 (10 SNPs out of a total of 182 SNPs) and the corresponding empirical *p* values obtained with “hapcc” and “hapccmax” (FAMHAP); reference is given about locus type, intragenic markers are highlighted (gray shading).

After validation by Monte-Carlo simulations with FAMHAP (c.f. 2.4.1), 5 SNPs remained significant at an α level of 0.05, tested with both “hapccmax” and “hapcc” methods (rs2242046, rs597354, rs717477, rs1860300, rs3625), and 2 SNPs (rs461311, rs1997660) remained significant at an α level of 0.05 for “hapcc” only. Supplementary Table S2-1 shows the *p* values of the χ^2 test for the 10 markers with *p* value < 0.05, and the corresponding empirical *p* values from the tests "hapcc" and "hapccmax" (FAMHAP). This set of SNPs

comprises 4 intragenic SNPs and 6 intergenic ones. In view of the number of SNPs tested, these results are not significant after Bonferroni correction.

Renynolds'D_R

Pairwise genetic distances between LUs were measured in the total sample (ALL) and in the native subset (GER) with the F_{ST} analogous Reynolds' D_R. The pairwise genetic distances are presented in the Supplementary Table S2-2.

**GER/ALL**	Aich	Alten	Augs	Ayst	Bob	Eur	Friedb	König	Langw	Neus	Pöttm	Rehl	Schwab
Aich	0.000	0.125	0.107	0.143	0.118	0.179	0.143	0.131	0.143	0.118	0.186	0.172	0.163
Alten	0.144	0.000	0.082	0.108	0.099	0.178	0.125	0.102	0.117	0.100	0.172	0.159	0.133
Augs	0.128	0.085	0.000	0.098	0.069	0.161	0.105	0.084	0.097	0.072	0.157	0.148	0.117
Ayst	0.169	0.126	0.115	0.000	0.114	0.185	0.133	0.120	0.120	0.110	0.180	0.184	0.149
Bob	0.142	0.107	0.082	0.139	0.000	0.175	0.121	0.099	0.118	0.100	0.171	0.157	0.134
Eur	0.192	0.177	0.162	0.195	0.182	0.000	0.190	0.176	0.183	0.176	0.221	0.217	0.196
Friedb	0.170	0.138	0.121	0.152	0.138	0.199	0.000	0.128	0.130	0.129	0.192	0.172	0.160
König	0.155	0.117	0.105	0.150	0.130	0.189	0.154	0.000	0.125	0.101	0.168	0.158	0.133
Langw	0.172	0.139	0.118	0.149	0.143	0.193	0.156	0.156	0.000	0.116	0.180	0.161	0.145
Neus	0.139	0.101	0.074	0.126	0.110	0.179	0.144	0.117	0.137	0.000	0.167	0.165	0.136
Pöttm	0.199	0.176	0.157	0.185	0.173	0.222	0.201	0.177	0.191	0.167	0.000	0.212	0.193
Rehl	0.187	0.171	0.155	0.202	0.170	0.221	0.184	0.172	0.180	0.172	0.220	0.000	0.170
Schwab	0.178	0.143	0.126	0.165	0.151	0.196	0.177	0.157	0.156	0.149	0.201	0.184	0.000

Supplementary Table S2-2 Matirx of pairwise D_R genetic distances between LUs (Aichach: Aich, Altenmünster: Alten, Augsburg: Augs, Aystetten: Ayst, Bobingen: Bob, Eurasburg: Eur, Friedberg: Friedb, Königsbrunn: König, Langweid: Langw, Neusäß: Neus, Pöttmes: Pöttm, Rehling: Rehl, Schwabmünchen: Schwab). The upper matrix section corresponds to the ALL data set. The lower matrix section correspond to the GER subset. The diagonal is indicated with gray shading.

The matrix of LU-pairwise Reynolds' genetic distance (D_R) was spatially analyzed taking Augsburg City as reference point. Landscapes were created with spatial interpolation (see Generation of Genetic Landscapes). On the data set ALL/LU13, areas with a higher percentage of samples with "land of birth"<>"Germany" showed lower D_R (Supp. Fig. S2-4a,c). This could be an indication of the higher degree of admixture of Augsburg city and its periphery in comparison to the peri-urban areas. In case of the subset of natives (GER/LU13, "land of birth"="Germany"), the surrounding ring of Augsburg City presented a significant lower D_R than the rest (fig. 4b) (R-statistics, package: wilcox.test, "Wilcoxon rank sum test with continuity correction", W = 2, p value = 0.01468). This may further indicate that a

sample which could be considered genetically homogeneous may still account for genetic substructures. The fact that the native population inhabiting areas with higher proportion of immigrants still differentiated from the rest peri-urban settlements may be interpreted as an indication that this population accounts for a higher degree of demographic admixture than the rest.

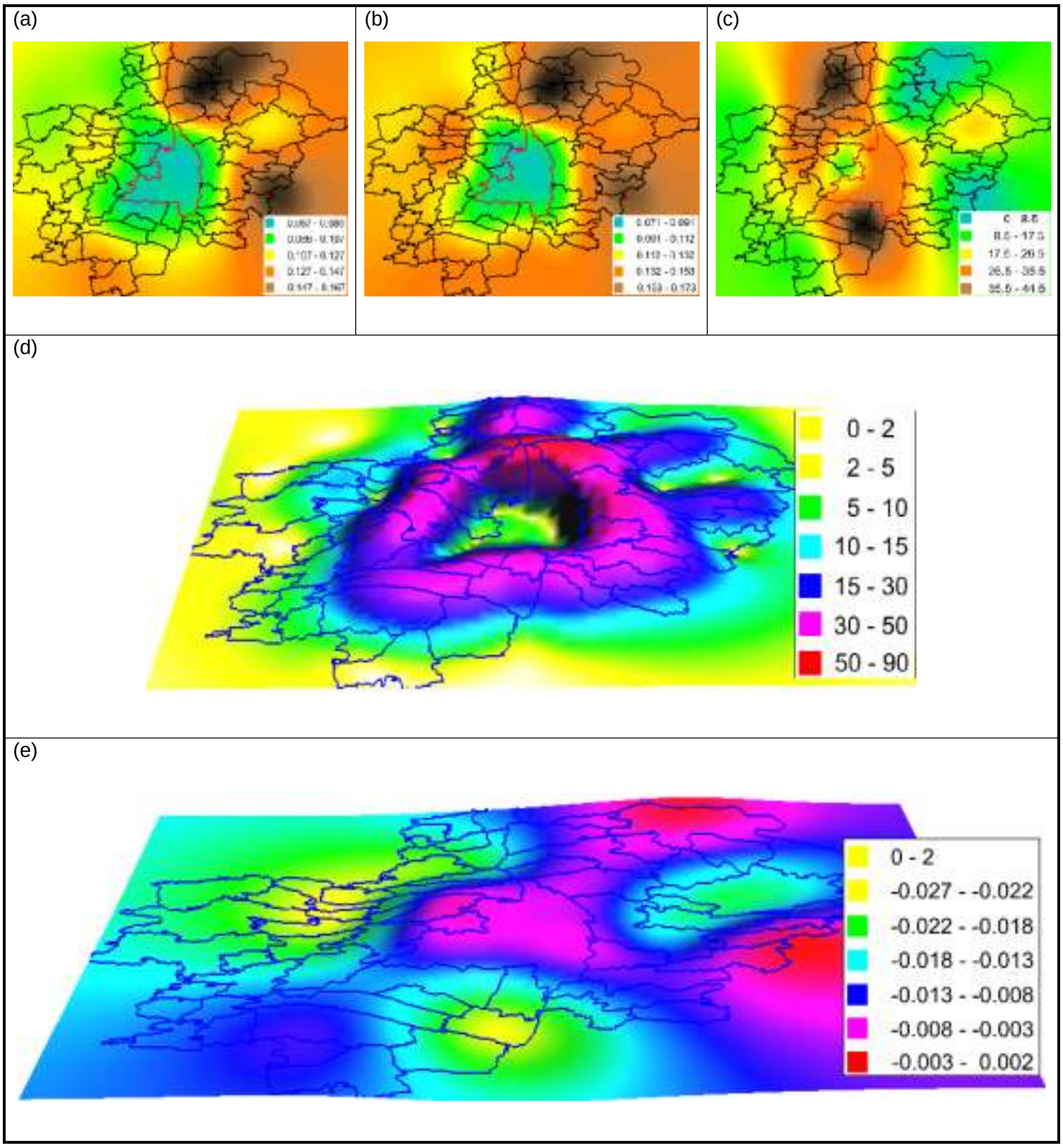

Supplementary Figure S2-4 Spatial pattern of Reynolds' D_R genetic distance to Augsburg (a) data set: ALL/LU13; (b) data set: GER/LU13; (c) Spatial frequency distribution of per cent of immigrants per LU ("land of birth<>"Germany"); (d) Gradient (slope) of the landscape delineated by the Reynolds' genetic distance to Augsburg (GER/LU13); (e) Differentiation between ALL/LU13 and GER/LU13 based on Reynolds' genetic distance to Augsburg.

The slope of this landscape, indicating the degree of change of the genetic distance to Augsburg City, showed a clear ring around the area of Augsburg City (Supp. Fig. S2-4d) and reflected the fast change of D_R values between urban periphery and countryside. Figure 4e displays the landscape of differences between the D_R values measured on the original sample (ALL/LU13) and on the reduced sample (GER/LU13). In the reduced sample (GER/LU13), distances increased everywhere except for the distant areas of Pöttmes and Eurasburg and the neighboring ones of Neusäß, Stadtbergen and Gersthofen.

This exploratory evaluation exposed a potential fine-scale genetic differentiation within a modern admixed population inhabiting a small area. It was observed that the peri-urban areas (the more countryside) showed a significant higher genetic distance (D_R) to Augsburg City than the Augsburg periphery. These results provided a further indication of fine-scale genetic differentiation in small areas as an effect of demographic factors. The failure of the χ^2 test for contingency tables to provide an indication of population substructure may indicate that this methods is too rough to search for fine-scale genetic patterns in small areas with reduced number of bi-allelic loci.

References

Becker T, Knapp M. 2004. Maximum-likelihood estimation of haplotype frequencies in nuclear families. Genet Epidemiol 27(1):21-32.

Becker T, Cichon S, Jonson E, Knapp M . 2005. Multiple testing in the context of haplotype analysis revisited: application to case-control data. Ann Hum Genet 69(Pt 6):747-56.

Reynolds J, Weir BS, Cockerham CC. Estimation of the Coancestry Coefficient. 1983. Basis for a Short-Term Genetic Distance. Genetics 105(3):767-79.

Weir BS. 1996. Genetic Data Analysis II. Sunderland; Sinauer Associates.

Wright S. 1951. The genetical structure of populations. Annals of Eugenics 15(4):323-54.

Supplement 3

Analysis of Genetic Differentiation of Urban vs Peri-Urban Areas

This analysis aimed assessing the relevance of genetic heterogeneity within a population for genetic association studies, since undetected genetic substructures may be one of the reasons for spurious or biased results. In a recent study (Steffens et al. 2006), it could be shown that even a minor degree of population stratification may be a possible source for confounding. The KORA S4 sample could not be clustered into different genetic subgroups using the software STRUCTURE (Pritchard et al. 2000), suggesting rather genetic homogeneity (Steffens et al. 2006). Since other studies have shown population substructure due to urban/rural factors (Vitart et al. 2005), we examined a potential differentiation between the most urban areas and the remaining peri-urban areas (some of these with a tendency to “quasi-rural” areas). We tested for genetic differentiation via χ^2 tests on the frequencies of the 212 genomic controls SNPs (Steffens et al. 2006) and calculation of the lambda inflation factor (median of χ^2 statistics divided by 1.386). Between the city of Augsburg (~260000 inhabitants) and all other communities (<30000) a lambda inflation factor of 1.043 with CI of [0.874, 1.309] could be found. Despite this low differentiation, a higher number of significant χ^2 test statistics have been observed as would have been expected under random distribution (Binomial test on portion of significant *p* values: *p*=0.038). Aggregating Augsburg and its adjacent communities in contrast with the countryside resulted in a slightly stronger differentiation of lambda=1.093 [0.877, 1.274] and *p*=0.021 for the respective Binomial test. These results indicate a small genetic heterogeneity due to an urban/peri-urban factor but with very minor relevance for genetic association analysis.

References

Pritchard JK, Stephens M, Donnelly P. 2000. Inference of Population Structure Using Multilocus Genotype Data. Genetics 155:945-959.

Steffens M, Lamina C, Illig T, et al. (27 co-authors). 2006. SNP-based analysis of genetic substructure in the German population. Hum Hered 62:20-29.

Vitart V, Carothers AD, Hayward C, Teague P, Hastie ND, Campbell H, Wright AF. 2005. Increased level of linkage disequilibrium in rural compared with urban communities: a factor to consider in association-study design. Am J Hum Genet 76:763-772.

Supplement 4

Sensitive Analysis of the Effect on Genetic Variation of Immigrants in the KORAS4 Survey

The effect of immigrants on total amount of genetic variation in the KORA S4 survey (integrated by randomly selected adult German citizens) was estimated applying the concept of genomic control (Devlin and Roeder 1999). This method proposes to estimate any inflation (λ) in the distribution of the association test statistics between unlinked genetic polymorphisms of the two considered groups (e.g. cases vs controls) generated by population structure based on the analysis of non-candidate loci. The *inflation factor*λ is computed as the ratio of the median of the Armitage's trend test statistics in relation to the expected 50% quantile of the association test χ^2 distribution (df=1) under the null hypothesis of no association between SNPs corresponding to the subsample of **immigrants** ("land of birth"<>"Germany") and the **natives** subset ("land of birth"="Germany"). A confidence interval for the *inflation factor* λ was computed using a bootstrapping procedure.

The H_0 hypothesis of equal genotype distribution in both groups (natives *vs* immigrants) was refused with a p= 0.03844256. The distribution of p values is presented in the supplementary figure S4. An inflation factor of λ= 1.169959 was estimated for the presence of immigrants in the ALL data set (ALL= natives + immigrants) and 95% confidence interval of 1.007788-1.416645 (distribution-free CI on the median based on the order statistics).

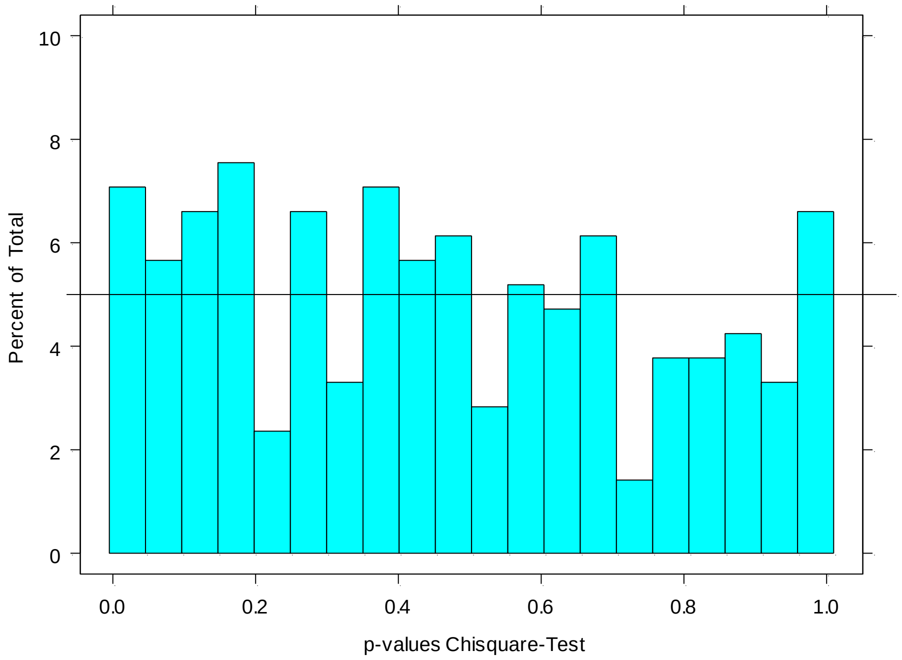

Supplementary Figure S4 Distribution of *p* values obtained with χ^2 test for differentiation of genotypes between immigrants and the native subset.

References

Devlin B, Roeder K. 1999. Genomic control for association studies. Biometrics 55:997–1004.

Weir BS. 1996. Genetic Data Analysis II. Sunderland; Sinauer Associates.

Supplement 5

Summary of Additional Multivariate Genetic Analyses

Several multivariate methods widely used to detect population genetic variation were used in order to analyze that the null hypothesis of a simple correlation of genetic distance with geographic distance does not fit the data. This included various implementations of the autocorrelation and Mantel test with varying parameters and models. Individual-based clustering methods were also applied to test for potential population substructure. A description of used methods is presented in **Multivariate Analysis of Spatial Population Structure**.

None of these tests provided indication of potential patterns of geographic variation in the study area. A brief result extract is presented here. Supp. Table S5 summarizes representative results obtained with SPAGeDI (Hardy and Vekemans 2002) and Supp. Figure S5 shows representative outputs of those obtained with PLINK! (Purcell et al. 2007) and EIGENSOFT (Patterson et al. 2006; Price et al. 2006). All together these results are a strong indication that other factors than simple isolation by distance (e.g. tested with SPAGeDI) or simple spatial population patterning (clustering or clinal) (e.g. evaluated with PLINK! and EIGENSOFT) may explain the fine-scale genetic diversity observed in the KORA S4 sample.

Distance classes	**1**	**2**	**3**	**4**	
Max distance	-1	0.0020	0.0039	0.0095	
Number of pairs	43076	78984	71183	71385	
% partic	99.9	98.4	100	100	
CV partic	0.9	0.68	0.75	0.93	
All loci	intra-group	2	3	4	**average**
Moran's *I* for individual allele frequency	0.00040	0.00000	0.00000	-0.00020	**0.00000**

Supplementary Table S5 Summary of the computed statistics with SPAGeDi 1.3 to quantify spatial correlation between genetic features and geographic coordinates; computations were conducted at INDIVIDUAL level (728 individuals, 206 autosomal SNP); larger number of distance classes resulted in higher values of CV (CV ≥ 1) [*number of pairs*: the number of pairwise comparisons belonging to the interval; *% partic*: proportion (%) of all individuals represented at least once in the interval; *CV partic*: coefficient of variation of the number of times each individual is represented]. Tests with further genetic measures and sample groupings provided similar outputs. Potential causes of the lack of indication of a spatial dependency with this tool is discussed in detailed in the section **Conclusions**.

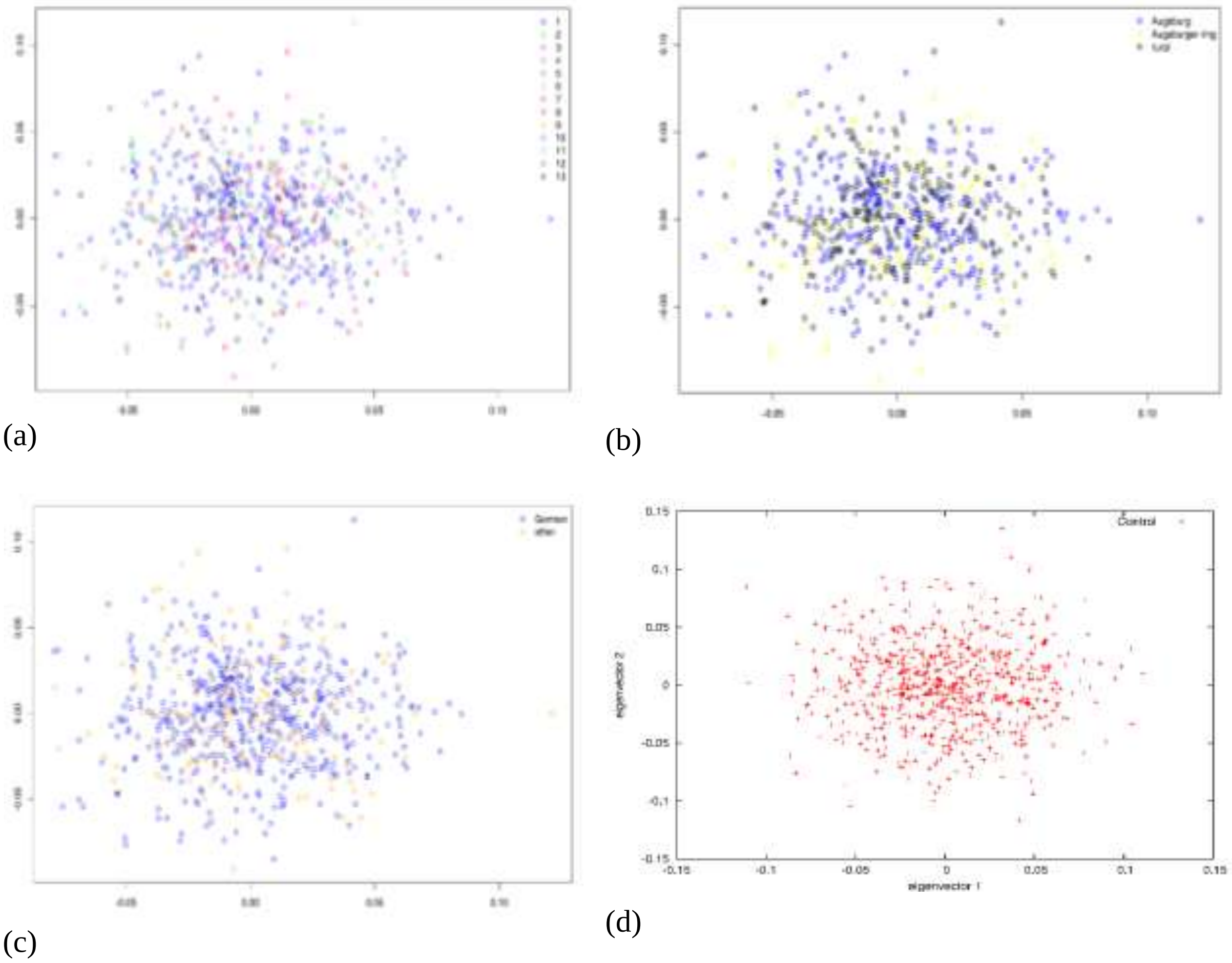

Supplementary Figure S5 Graphical outputs of tests used to detect potential population substructure using PLINK! (a-c) and EIGENSOFT (d) based on 728 individuals (367 males, 361 females) and 206 autosomal SNPs. (a-c) PLINK! output run with default options: (a) samples were differentiated according to LU (1-13); (b) samples were differentiated according to the location of LU in reference to the distance to Augsburg City; (c) samples were differentiated according to the land of birth. (d) EIGENSOFT output, run with default options, showing the distribution of individuals according to the top two Principal Components.

References

Hardy OJ, Vekemans X. 2002. SPAGeDi: a versatile computer program to analyse spatial genetic structure at the individual or population levels. Molecular Ecology Notes 2: 618-620.

Patterson N, Price AL, Reich D. 2006. Population structure and eigenanalysis. PLoS Genet 2:e190.

Price AL, Patterson NJ, Plenge RM, Weinblatt ME, Shadick NA, Reich D. 2006. Principal components analysis corrects for stratification in genome-wide association studies. Nat Genet 38:904-909.

Purcell S, Neale B, Todd-Brown K, Thomas L, Ferreira MA, Bender D, Maller J, Sklar P, de Bakker PI, Daly MJ, Sham PC. 2007. PLINK: a tool set for whole-genome association and population-based linkage analyses. Am J Hum Genet 81:559-575.

Supplement 6

The KORA S4 Marker Set

SNP	Chr	Position	Region	Alleles	minor	Genotypes	MAF	95%-CI	*p* HWE (χ^2)
rs1000336	20	40034662	Intergenic	A/G	G	(453,242,030)	0.208	[0.187, 0.229]	0.744
rs1001238	2	179290033	Intragenic	A/G	G	(427,246,031)	0.219	[0.197, 0.240]	0.553
rs1001544	17	51510198	Intergenic	C/G	G	(343,310,069)	0.310	[0.286, 0.334]	0.931
rs1002202	3	70953780	Intergenic	C/T	T	(214,373,130)	0.441	[0.416, 0.466]	0.141
rs1008350	20	37383594	Intergenic	C/G	C	(161,335,181)	0.485	[0.458, 0.512]	0.805
rs1013024	1	98531558	Intergenic	A/G	A	(062,304,331)	0.307	[0.283, 0.331]	0.510
rs1014863	20	12767817	Intergenic	C/T	C	(041,258,425)	0.235	[0.213, 0.257]	0.823
rs1015558	23	22890257	Intergenic	A/C	A	(035,057,633)	0.088	[0.070, 0.105]	n.a.
rs1016029	4	13473802	Intergenic	A/C	C	(389,273,044)	0.256	[0.233, 0.278]	0.671
rs1020298	17	50109444	Intergenic	A/G	G	(295,328,104)	0.369	[0.343, 0.394]	0.407
rs1021670	12	24127499	Intergenic	A/G	A	(068,293,356)	0.299	[0.275, 0.323]	0.495
rs1021704	5	21056492	Intergenic	A/G	A	(042,287,387)	0.259	[0.237, 0.281]	0.238
rs1021711	5	31020056	Intergenic	G/T	T	(271,312,136)	0.406	[0.379, 0.433]	0.007
rs1022565	20	41899953	Intergenic	A/G	A	(010,178,521)	0.140	[0.122, 0.157]	0.232
rs1024818	2	67174828	Intergenic	C/T	T	(318,279,087)	0.331	[0.305, 0.357]	0.038
rs1025776	2	35394043	Intergenic	A/C	C	(261,330,126)	0.406	[0.380, 0.432]	0.221
rs1026937	18	33874889	Intergenic	A/C	A	(163,324,193)	0.478	[0.451, 0.505]	0.239
rs1029135	10	85413965	Intergenic	C/T	T	(358,294,059)	0.290	[0.266, 0.313]	0.901
rs1034489	2	120601540	Intragenic	C/T	C	(047,275,382)	0.262	[0.239, 0.285]	0.792
rs1036268	10	132580441	Intergenic	G/T	G	(080,330,315)	0.338	[0.314, 0.362]	0.643
rs1042917	21	46370196	Intragenic	A/G	G	(190,337,178)	0.491	[0.465, 0.518]	0.246
rs1045002	14	54888270	Intragenic	A/T	A	(101,340,259)	0.387	[0.362, 0.412]	0.533
rs1046276	16	30822127	Intragenic	C/T	T	(300,320,084)	0.347	[0.322, 0.371]	0.925
rs1056513	1	62092319	Intragenic	A/G	G	(296,315,095)	0.358	[0.332, 0.383]	0.442
rs1056522	3	127744043	Intragenic	C/T	T	(349,273,061)	0.289	[0.265, 0.314]	0.469
rs1061472	13	51422489	Intragenic	A/G	A	(132,347,227)	0.433	[0.407, 0.459]	0.976
rs1074242	14	84293606	Intergenic	A/C	A	(010,137,574)	0.109	[0.093, 0.125]	0.577
rs1074670	9	71246327	Intergenic	A/G	G	(207,350,163)	0.469	[0.443, 0.496]	0.517
rs10842971	12	9194563	Intragenic	A/T	T	(372,262,065)	0.280	[0.256, 0.305]	0.060
rs11096957	4	38599057	Intragenic	A/C	C	(271,348,085)	0.368	[0.343, 0.392]	0.096
rs1157573	23	140584162	Intergenic	A/C	A	(083,119,519)	0.198	[0.173, 0.223]	n.a.
rs12529	10	5126651	Intragenic	C/G	G	(230,358,116)	0.419	[0.394, 0.444]	0.238
rs12876018	13	95338205	Intragenic	G/T	G	(114,359,235)	0.415	[0.389, 0.440]	0.235
rs1316515	1	80830006	Intergenic	A/G	A	(000,046,665)	0.032	[0.023, 0.041]	0.373
rs1322296	9	10622009	Intergenic	C/T	T	(608,109,004)	0.081	[0.067, 0.095]	0.709
rs1328994	9	32707013	Intergenic	A/G	A	(129,342,252)	0.415	[0.389, 0.441]	0.489
rs1329056	9	117461088	Intergenic	A/C	A	(025,235,451)	0.200	[0.180, 0.221]	0.405
rs1335995	10	33070114	Intergenic	A/G	G	(612,103,004)	0.077	[0.063, 0.091]	0.882
rs1338799	10	57299685	Intergenic	A/G	G	(269,349,100)	0.382	[0.358, 0.407]	0.435
rs1345829	5	100375500	Intergenic	G/T	G	(093,310,317)	0.344	[0.319, 0.370]	0.211

rs1346859	2	82236631	Intergenic	C/T	T	(443,232,040)	0.218	[0.196, 0.240]	0.191
rs1350401	6	77426097	Intergenic	A/T	A	(036,276,405)	0.243	[0.221, 0.264]	0.206
rs1354004	4	45171130	Intergenic	A/G	A	(062,279,366)	0.285	[0.261, 0.309]	0.399
rs1365084	11	43066630	Intergenic	A/G	G	(360,289,070)	0.298	[0.274, 0.322]	0.284
rs1379736	8	87873425	Intergenic	C/T	T	(419,266,037)	0.235	[0.214, 0.257]	0.531
rs1385934	7	46041312	Intergenic	G/T	T	(216,301,163)	0.461	[0.433, 0.489]	0.004
rs1385984	4	72378710	Intergenic	A/G	G	(513,195,011)	0.151	[0.133, 0.169]	0.118
rs1388294	4	100990419	Intergenic	C/T	T	(722,002,000)	0.001	[0.000, 0.003]	0.970
rs1423639	16	25553093	Intergenic	A/T	A	(028,199,486)	0.179	[0.158, 0.199]	0.185
rs1425174	11	29897578	Intergenic	C/T	T	(258,332,126)	0.408	[0.382, 0.434]	0.284
rs1436394	16	5764497	Intergenic	A/G	G	(400,256,066)	0.269	[0.245, 0.293]	0.009
rs1486737	16	53733937	Intergenic	C/T	T	(458,234,032)	0.206	[0.185, 0.227]	0.761
rs148939	16	76225851	Intergenic	C/T	C	(037,267,361)	0.256	[0.234, 0.279]	0.172
rs1502812	12	56852627	Intergenic	A/G	A	(048,252,419)	0.242	[0.219, 0.265]	0.231
rs1505279	15	37325165	Intergenic	C/T	C	(089,323,312)	0.346	[0.321, 0.371]	0.702
rs1520431	15	44163734	Intergenic	C/T	C	(123,367,237)	0.422	[0.397, 0.447]	0.344
rs1524238	7	10205181	Intergenic	A/T	A	(093,322,307)	0.352	[0.327, 0.377]	0.552
rs1524760	7	123834235	Intergenic	C/T	T	(375,290,052)	0.275	[0.252, 0.298]	0.690
rs1530242	4	35157039	Intergenic	A/G	A	(066,314,339)	0.310	[0.286, 0.334]	0.581
rs1538279	6	18935284	Intergenic	C/T	C	(061,333,326)	0.316	[0.293, 0.339]	0.061
rs155320	18	10949495	Intergenic	A/G	A	(186,340,189)	0.498	[0.471, 0.524]	0.191
rs1561419	20	58571373	Intergenic	C/G	C	(092,330,303)	0.354	[0.330, 0.379]	0.884
rs1570043	20	22254243	Intergenic	C/G	C	(066,294,361)	0.295	[0.272, 0.319]	0.582
rs1571363	9	26430477	Intergenic	C/T	C	(001,047,664)	0.034	[0.025, 0.044]	0.859
rs1572583	10	113430035	Intergenic	A/C	C	(273,335,114)	0.390	[0.364, 0.415]	0.506
rs171603	16	9375312	Intergenic	C/T	C	(035,230,457)	0.208	[0.186, 0.229]	0.386
rs1731017	16	8747455	Intragenic	C/T	T	(256,353,092)	0.383	[0.358, 0.408]	0.083
rs1760897	14	19946093	Intragenic	C/T	C	(071,311,321)	0.322	[0.298, 0.346]	0.733
rs1801224	10	17187527	Intragenic	A/C	C	(317,306,070)	0.322	[0.297, 0.346]	0.759
rs1860300	17	11075412	Intergenic	A/C	C	(250,350,124)	0.413	[0.388, 0.438]	0.937
rs1874243	7	67272732	Intergenic	C/T	C	(136,320,264)	0.411	[0.385, 0.438]	0.028
rs1883848	20	60998751	Intragenic	A/G	G	(259,310,107)	0.388	[0.361, 0.414]	0.377
rs1884517	22	46646084	Intergenic	A/G	A	(059,252,311)	0.297	[0.272, 0.323]	0.446
rs1923626	1	174488161	Intergenic	A/G	G	(234,346,135)	0.431	[0.405, 0.457]	0.723
rs1926119	23	94921762	Intergenic	A/C	C	(534,116,073)	0.181	[0.157, 0.205]	n.a.
rs1935384	9	101789897	Intergenic	C/G	G	(556,140,026)	0.133	[0.114, 0.152]	0.000
rs1945906	11	81238725	Intergenic	G/T	G	(068,313,338)	0.312	[0.288, 0.336]	0.716
rs1946677	2	153748313	Intergenic	C/T	T	(182,375,161)	0.485	[0.460, 0.511]	0.223
rs1947743	11	96975551	Intergenic	C/T	C	(123,329,255)	0.407	[0.381, 0.433]	0.343
rs1995641	3	44918393	Intragenic	A/G	A	(124,342,240)	0.418	[0.392, 0.444]	0.909
rs1997660	6	28377642	Intragenic	C/T	C	(055,313,334)	0.301	[0.278, 0.325]	0.118
rs2000250	14	62054719	Intergenic	G/T	G	(171,384,172)	0.499	[0.474, 0.524]	0.128
rs2014269	3	135895510	Intergenic	A/G	G	(339,318,069)	0.314	[0.290, 0.338]	0.654
rs2014790	5	25208956	Intergenic	A/G	G	(520,179,017)	0.149	[0.130, 0.167]	0.732
rs2021952	23	146385382	Intergenic	G/T	G	(028,058,641)	0.078	[0.062, 0.095]	n.a.
rs2031549	6	9363165	Intergenic	C/G	C	(028,244,437)	0.212	[0.191, 0.233]	0.400
rs2034127	3	59343114	Intergenic	A/G	A	(021,190,514)	0.160	[0.141, 0.179]	0.500

rs2037814	2	73587324	Intragenic	A/C	A	(015,168,514)	0.142	[0.124, 0.160]	0.771
rs2070132	19	41419205	Intragenic	A/G	A	(116,341,248)	0.406	[0.381, 0.432]	0.947
rs2076740	8	134053240	Intragenic	C/T	T	(287,325,092)	0.362	[0.336, 0.387]	1.000
rs2173904	9	2818765	Intragenic	C/G	C	(132,355,218)	0.439	[0.413, 0.465]	0.554
rs220263	21	42355230	Intergenic	A/G	G	(233,354,135)	0.432	[0.407, 0.458]	0.979
rs2227275	20	43359987	Intragenic	A/G	A	(047,259,394)	0.252	[0.229, 0.275]	0.617
rs2232700	14	93826203	Intragenic	A/T	T	(499,182,023)	0.162	[0.142, 0.182]	0.207
rs2235079	23	125555222	Intergenic	A/G	A	(187,172,368)	0.376	[0.345, 0.406]	n.a.
rs2239359	16	88376981	Intragenic	C/T	T	(281,301,122)	0.387	[0.360, 0.414]	0.009
rs2242046	15	83279733	Intragenic	C/T	C	(171,340,193)	0.484	[0.458, 0.511]	0.379
rs2250242	9	114183527	Intragenic	A/G	G	(183,350,171)	0.491	[0.465, 0.518]	0.886
rs2261988	19	4861889	Intragenic	A/C	A	(093,302,305)	0.349	[0.323, 0.374]	0.186
rs2274223	10	96056331	Intragenic	A/G	G	(301,325,080)	0.343	[0.319, 0.368]	0.582
rs2274327	1	8943672	Intragenic	C/T	T	(262,305,140)	0.414	[0.387, 0.441]	0.003
rs2275799	10	115399830	Intragenic	A/G	A	(049,270,386)	0.261	[0.238, 0.284]	0.849
rs2289025	2	205794726	Intragenic	A/G	G	(258,330,110)	0.394	[0.368, 0.420]	0.793
rs2289043	4	96463500	Intragenic	C/T	T	(353,298,054)	0.288	[0.265, 0.311]	0.413
rs2302147	7	155968609	Intragenic	C/G	C	(087,315,302)	0.347	[0.322, 0.372]	0.729
rs2302190	17	53939507	Intragenic	C/T	C	(035,231,433)	0.215	[0.194, 0.237]	0.561
rs2465811	12	69276321	Intragenic	C/T	C	(058,291,353)	0.290	[0.266, 0.314]	0.856
rs25433	18	2298472	Intergenic	C/G	C	(091,340,288)	0.363	[0.338, 0.388]	0.546
rs263842	13	54459287	Intergenic	C/T	C	(010,082,574)	0.077	[0.061, 0.092]	0.001
rs2658658	12	51075195	Intragenic	A/G	A	(142,354,211)	0.451	[0.425, 0.477]	0.769
rs2725362	8	31118822	Intragenic	G/T	T	(219,348,139)	0.443	[0.417, 0.469]	0.972
rs272893	5	131690961	Intragenic	A/G	A	(102,320,282)	0.372	[0.347, 0.398]	0.468
rs2824790	21	18677911	Intragenic	C/G	G	(369,281,055)	0.277	[0.254, 0.301]	0.882
rs30386	5	179223451	Intragenic	A/C	C	(196,335,172)	0.483	[0.456, 0.510]	0.224
rs315427	6	153607668	Intergenic	A/G	A	(007,096,601)	0.078	[0.064, 0.093]	0.157
rs318373	5	143300064	Intergenic	C/T	C	(168,339,217)	0.466	[0.440, 0.493]	0.111
rs3195676	5	34043857	Intragenic	A/G	G	(201,342,159)	0.470	[0.444, 0.497]	0.557
rs328418	4	187933042	Intragenic	A/G	A	(143,350,211)	0.452	[0.426, 0.478]	0.922
rs345182	17	55595546	Intergenic	A/G	G	(457,240,029)	0.205	[0.185, 0.226]	0.719
rs3625	14	74974413	Intragenic	A/G	A	(156,348,188)	0.477	[0.451, 0.503]	0.835
rs3746731	20	23013209	Intragenic	A/G	G	(239,327,138)	0.428	[0.402, 0.455]	0.172
rs3754112	1	117266463	Intragenic	C/T	C	(085,310,311)	0.340	[0.315, 0.365]	0.567
rs3809982	18	54354054	Intragenic	C/T	T	(177,347,166)	0.492	[0.466, 0.518]	0.874
rs3811740	4	129164824	Intragenic	A/T	A	(077,305,321)	0.326	[0.302, 0.351]	0.722
rs389783	19	36942225	Intergenic	G/T	T	(474,216,033)	0.195	[0.174, 0.216]	0.192
rs39489	7	117745406	Intergenic	A/G	G	(322,316,080)	0.331	[0.307, 0.356]	0.852
rs4379869	11	76315299	Intragenic	A/G	G	(397,249,062)	0.263	[0.239, 0.287]	0.013
rs438034	1	211219012	Intragenic	C/T	T	(207,334,164)	0.470	[0.443, 0.496]	0.194
rs444772	8	55701610	Intragenic	A/G	A	(049,285,372)	0.271	[0.248, 0.294]	0.575
rs450015	18	7154754	Intergenic	C/T	C	(130,364,227)	0.433	[0.408, 0.458]	0.447
rs461311	1	112367036	Intergenic	G/T	G	(041,269,408)	0.244	[0.222, 0.267]	0.701
rs4918	3	187821084	Intragenic	C/G	G	(322,320,061)	0.314	[0.291, 0.338]	0.138
rs548146	11	110541266	Intergenic	C/T	C	(080,309,336)	0.323	[0.299, 0.348]	0.481
rs558912	2	16912881	Intergenic	C/T	T	(277,342,105)	0.381	[0.356, 0.406]	0.973

rs5759598	22	21805516	Intragenic	G/T	T	(246,334,123)	0.413	[0.387, 0.439]	0.600
rs591120	1	174634410	Intragenic	C/G	C	(133,331,242)	0.423	[0.397, 0.449]	0.295
rs597354	13	85082205	Intergenic	A/G	G	(507,202,017)	0.163	[0.144, 0.181]	0.552
rs6572	22	35945945	Intragenic	C/G	G	(210,345,138)	0.448	[0.422, 0.474]	0.863
rs6591561	11	59826752	Intragenic	A/G	G	(364,275,065)	0.288	[0.263, 0.312]	0.214
rs663528	13	29505076	Intergenic	A/C	A	(161,359,202)	0.472	[0.446, 0.497]	0.950
rs676210	2	21143176	Intragenic	A/G	A	(031,233,436)	0.211	[0.189, 0.232]	0.985
rs679620	11	102218830	Intragenic	A/G	G	(193,353,158)	0.475	[0.449, 0.501]	0.888
rs701616	1	205046263	Intergenic	A/G	A	(138,335,249)	0.423	[0.397, 0.449]	0.183
rs705993	8	70314229	Intergenic	A/G	G	(419,263,040)	0.238	[0.216, 0.259]	0.880
rs709029	20	5613404	Intergenic	C/T	T	(486,191,026)	0.173	[0.153, 0.193]	0.187
rs709564	3	108579237	Intragenic	A/G	A	(042,246,415)	0.235	[0.212, 0.257]	0.492
rs715437	3	145336324	Intergenic	C/T	C	(064,276,380)	0.281	[0.257, 0.304]	0.176
rs7158	9	37772111	Intragenic	C/T	C	(165,363,176)	0.492	[0.466, 0.518]	0.403
rs717218	8	41339319	Intergenic	C/T	T	(227,363,134)	0.436	[0.410, 0.461]	0.598
rs717477	6	40264528	Intergenic	C/T	T	(357,303,062)	0.296	[0.272, 0.319]	0.840
rs718564	14	81121578	Intergenic	A/G	G	(335,311,078)	0.323	[0.298, 0.347]	0.647
rs718793	4	27702586	Intergenic	C/G	G	(396,273,040)	0.249	[0.227, 0.271]	0.429
rs719354	16	71600430	Intergenic	C/T	T	(490,196,032)	0.181	[0.160, 0.202]	0.033
rs719437	6	23614456	Intergenic	A/G	G	(358,313,050)	0.286	[0.264, 0.309]	0.096
rs720487	7	90687868	Intergenic	C/T	C	(073,333,315)	0.332	[0.308, 0.356]	0.271
rs725317	13	104028615	Intergenic	A/G	A	(058,319,344)	0.302	[0.279, 0.325]	0.178
rs725747	23	43006456	Intergenic	C/T	C	(194,162,366)	0.381	[0.350, 0.412]	n.a.
rs727321	16	63281880	Intergenic	C/T	T	(175,341,162)	0.490	[0.464, 0.517]	0.870
rs727811	6	165015745	Intergenic	A/C	C	(228,345,147)	0.444	[0.418, 0.470]	0.430
rs728089	14	37282250	Intergenic	C/T	C	(085,290,334)	0.324	[0.299, 0.350]	0.075
rs729333	10	8927864	Intergenic	A/G	A	(088,360,268)	0.374	[0.350, 0.398]	0.049
rs730899	3	116408505	Intergenic	C/T	C	(147,352,220)	0.449	[0.423, 0.475]	0.775
rs7313	7	28843449	Intragenic	C/T	T	(223,356,126)	0.431	[0.406, 0.457]	0.435
rs7323	13	26907031	Intragenic	C/G	C	(064,256,384)	0.273	[0.248, 0.297]	0.027
rs733036	15	91490197	Intergenic	A/G	G	(600,123,004)	0.090	[0.076, 0.105]	0.390
rs734204	19	43231828	Intergenic	C/T	C	(073,283,368)	0.296	[0.272, 0.321]	0.092
rs735309	12	68858684	Intergenic	A/G	G	(336,311,058)	0.303	[0.279, 0.326]	0.235
rs737622	22	27202269	Intergenic	A/C	C	(219,352,143)	0.447	[0.421, 0.473]	0.942
rs739226	22	25540980	Intergenic	A/G	A	(059,308,351)	0.297	[0.273, 0.320]	0.454
rs745181	11	11305404	Intergenic	A/G	G	(307,336,083)	0.346	[0.322, 0.370]	0.535
rs753653	5	127000709	Intergenic	C/T	T	(494,216,016)	0.171	[0.152, 0.190]	0.175
rs754027	18	4630454	Intergenic	A/T	A	(000,065,663)	0.045	[0.034, 0.055]	0.207
rs758326	19	35417874	Intergenic	A/G	A	(036,222,406)	0.221	[0.199, 0.244]	0.436
rs759944	8	129856608	Intergenic	A/G	G	(224,361,142)	0.444	[0.418, 0.469]	0.873
rs763926	22	33664590	Intergenic	A/C	A	(077,284,361)	0.303	[0.279, 0.328]	0.063
rs768352	2	185155197	Intergenic	A/G	A	(177,346,184)	0.495	[0.469, 0.521]	0.574
rs768365	10	9737760	Intergenic	C/T	T	(185,367,175)	0.493	[0.468, 0.519]	0.791
rs768703	9	18060475	Intergenic	G/T	T	(228,341,157)	0.451	[0.425, 0.477]	0.165
rs769295	1	221636371	Intergenic	C/T	C	(000,043,652)	0.031	[0.022, 0.040]	0.400
rs773837	19	16743421	Intergenic	A/C	C	(665,057,001)	0.041	[0.031, 0.051]	0.847
rs7978353	12	121142869	Intragenic	A/G	G	(277,328,100)	0.374	[0.349, 0.400]	0.855

rs803064	7	101510956	Intragenic	C/T	C	(123,353,229)	0.425	[0.399, 0.450]	0.514
rs871644	1	18042994	Intergenic	C/T	T	(429,249,030)	0.218	[0.197, 0.239]	0.413
rs878830	11	125815292	Intragenic	A/T	T	(339,301,065)	0.306	[0.282, 0.330]	0.877
rs881003	11	131110537	Intergenic	A/G	A	(019,219,483)	0.178	[0.159, 0.198]	0.321
rs888231	9	125906345	Intergenic	A/C	A	(115,323,289)	0.380	[0.355, 0.406]	0.122
rs889350	19	34089180	Intergenic	A/G	A	(092,325,309)	0.351	[0.326, 0.375]	0.650
rs890033	8	6010779	Intergenic	C/G	G	(465,222,039)	0.207	[0.185, 0.228]	0.070
rs892586	18	42814427	Intragenic	G/T	G	(171,322,206)	0.475	[0.448, 0.502]	0.043
rs896664	15	68028239	Intergenic	A/T	T	(395,271,058)	0.267	[0.244, 0.291]	0.233
rs897136	2	221064145	Intergenic	C/T	C	(031,270,425)	0.229	[0.208, 0.250]	0.143
rs901063	17	76210291	Intergenic	A/T	T	(575,137,012)	0.111	[0.095, 0.128]	0.251
rs901089	3	32057793	Intergenic	C/T	C	(025,205,488)	0.178	[0.158, 0.198]	0.547
rs906882	11	24599163	Intergenic	C/T	T	(290,343,088)	0.360	[0.336, 0.384]	0.383
rs919308	5	66604140	Intergenic	A/G	G	(312,323,079)	0.337	[0.312, 0.361]	0.736
rs920287	5	13081530	Intergenic	A/C	C	(248,355,120)	0.411	[0.386, 0.437]	0.711
rs922082	21	23504521	Intergenic	G/T	T	(528,171,015)	0.141	[0.123, 0.159]	0.792
rs925760	8	115297043	Intergenic	G/T	T	(182,357,181)	0.499	[0.473, 0.525]	0.823
rs927470	20	19059511	Intergenic	C/T	T	(306,310,095)	0.352	[0.326, 0.377]	0.243
rs930706	8	35097494	Intergenic	C/T	T	(231,364,128)	0.429	[0.404, 0.454]	0.455
rs934472	9	18765810	Intragenic	A/C	C	(191,342,172)	0.487	[0.460, 0.513]	0.440
rs937669	8	31806478	Intergenic	C/G	C	(027,249,437)	0.212	[0.192, 0.233]	0.245
rs937925	1	159959809	Intergenic	A/G	G	(624,091,006)	0.071	[0.058, 0.085]	0.192
rs9381594	6	47757653	Intragenic	A/G	A	(091,318,292)	0.357	[0.331, 0.382]	0.762
rs961598	18	53008493	Intergenic	G/T	G	(057,290,373)	0.281	[0.257, 0.304]	0.952
rs966204	18	67864849	Intergenic	C/T	C	(070,282,275)	0.337	[0.310, 0.363]	0.857
rs971824	18	59863837	Intergenic	C/T	T	(425,254,042)	0.234	[0.212, 0.256]	0.620
rs982448	16	26449596	Intergenic	A/G	G	(379,242,036)	0.239	[0.216, 0.262]	0.745
rs991068	12	82762113	Intergenic	A/G	G	(304,307,109)	0.365	[0.339, 0.390]	0.032
rs998239	10	4534374	Intergenic	G/T	T	(402,273,041)	0.248	[0.226, 0.270]	0.547
rs999318	4	167007810	Intergenic	C/T	C	(058,293,370)	0.284	[0.260, 0.307]	1.000
rs999972	12	23467867	Intergenic	A/G	A	(024,206,490)	0.176	[0.157, 0.196]	0.682

Supplementary Table S6 Genetic properties of the KORA S4 marker set[1]. Giver are the rs number, chromosome, chromosomal position in base pairs, the alleles, the minor allele, counts of genotype observed in the sample, minor allele frequency (MAF) and the 95% confidence intervals as well as the *p* values of Hardy-Weinberg deviation (uncorrected) of each marker.

[1] Information based on NCBI dbSNP Build 123 (Year 2004).

Supplement 7

Genetic Relatedness

The graphic tool GRR (Graphical Representation of Relationships; Abecasis et al. 2001) was used to test for potential relationship between individuals of the KORA S4 sample. This program estimates the biological relationship of all pair of individuals of a sample based on amount of alleles shared over all loci [identical by state (IBS)].

Results indicate that all pairs of individuals are similar in respect to the proportion of shared alleles, which is consistent with a sample of unrelated individuals. No indication of undocumented biological relationship was found with this tool.

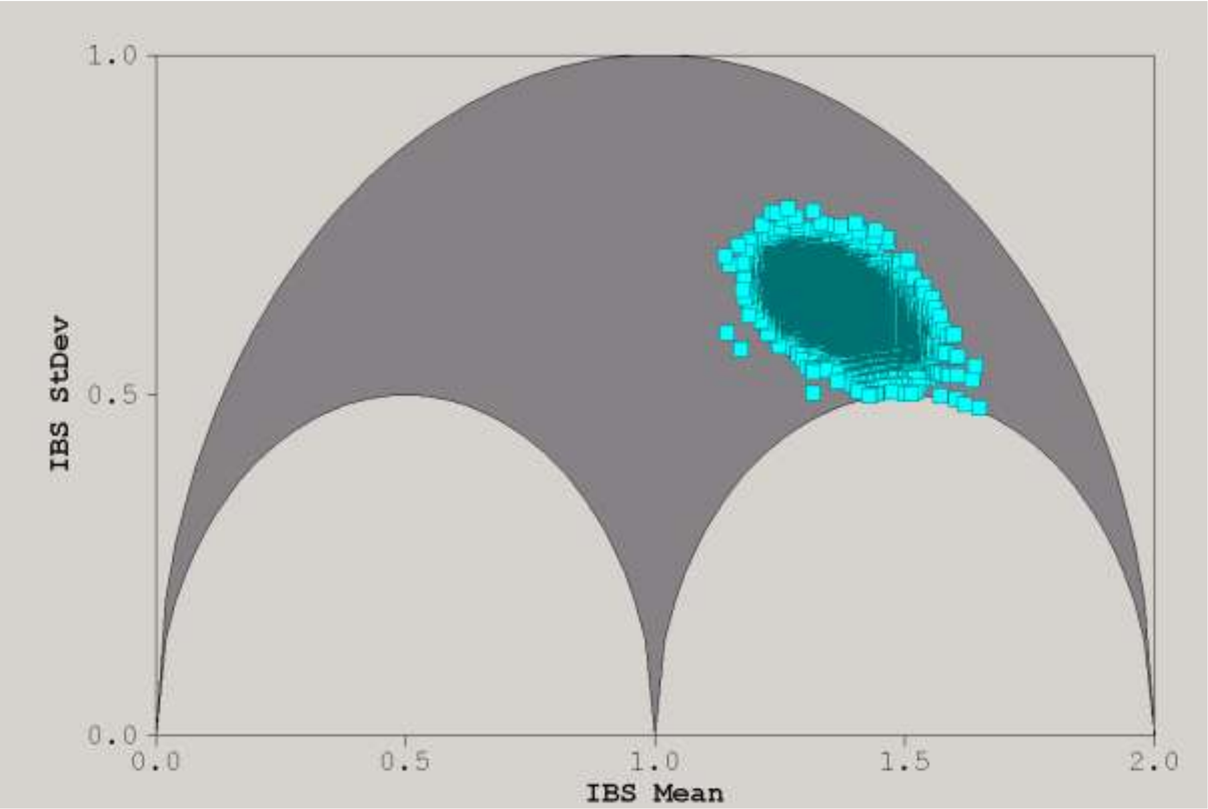

Supplementary Figure S7 Estimation of the relationship of pairs of individuals of the KORA S4 sample.

References

Abecasis GR1, Cherny SS, Cookson WO, Cardon LR. 2001. GRR: graphical representation of relationship errors. Bioinformatics 17(8):742-3.
